# Gut Bacterial and Fungal Communities of the Tibetan Eared Pheasant (*Crossoptilon harmani*) Across a Captive–Temple–Wild Gradient: Implications for Ex Situ Conservation

**DOI:** 10.3390/ani16213414

**Published:** 2026-10-29

**Authors:** Cong Wei, Shiyang Weng, Yanchao Liu, Jianchuan Li

**Affiliations:** 1 Key Laboratory of Biological Resources and Biosecurity of Xizang Autonomous Region, Institute of Plateau Biology of Xizang Autonomous Region, Lhasa 850001, China; 2 Natural Science Museum of Xizang, Lhasa 850001, China

**Keywords:** *Crossoptilon harmani*, gut microbiota, 16S rRNA, ITS, captive–wild comparison, ex situ conservation, bacterial–fungal differential response

## Abstract

Gut microbiota influence nutrient acquisition, immune function, and pathogen resistance, yet are rarely considered in ex situ planning. We compared gut bacterial (16S ribosomal RNA; 16S rRNA) and fungal (internal transcribed spacer 1; ITS1) communities of the Tibetan eared pheasant (*Crossoptilon harmani*), a protected Galliform endemic to the Qinghai–Tibet Plateau, across a management gradient: fully captive (*n* = 3), wild (*n* = 7), and free-ranging monastery birds receiving supplementary barley (*n* = 6); samples were not linked to identified donors, so the kingdoms are compared at group level only. Monastery birds had the numerically highest bacterial Shannon diversity (5.79), above wild (5.54) and captive birds (2.93), although the among-group test was not significant (*p* = 0.13). Permutational multivariate analysis of variance (PERMANOVA) showed significant bacterial differentiation (Bray–Curtis *R*^2^ = 0.309, *p* = 0.001), driven by the captive group, which showed *Lactobacillus* dominance (51.4%). Fungal communities also differed significantly (*R*^2^ = 0.194, *p* = 0.008), with no compositional turnover (Jaccard *p* = 0.447). Birds in natural habitat with supplementary feeding were no more dissimilar from wild birds than wild birds were among themselves, whereas fully captive birds were clearly separated; this pattern warrants testing in replicated, multi-site studies.

## Simple Summary

The microbes living in an animal’s gut influence its nutrition and immunity, yet they are rarely considered when planning how to care for protected species in captivity. We studied the gut bacteria and fungi of the Tibetan eared pheasant, a protected bird from the Qinghai–Tibet Plateau, under three living conditions: fully captive, wild, and free-ranging near a temple where monks offer extra barley. Captive birds had much simpler gut bacterial communities dominated by a single bacterial group, whereas temple and wild birds had much richer communities. Gut fungi changed far less than bacteria across all conditions. These results suggest that birds in near-natural settings with extra feeding had a gut bacterial community no more dissimilar from wild birds than wild birds were from one another, whereas fully captive birds were clearly separated. This is a hypothesis for ex situ management rather than a demonstrated conservation benefit.

## 1. Introduction

Understanding how different management strategies shape host-associated microbial communities is an emerging frontier in conservation biology. Gut microbiota influence nutrient acquisition, immune function, and pathogen resistance, yet are rarely considered in ex situ management planning. Birds under different living conditions, from full captivity to free-ranging, experience contrasting diets, social densities, and environmental exposures, each of which may restructure their gut microbial communities with potential consequences for host fitness. The Tibetan eared pheasant (*Crossoptilon harmani*), a phasianid endemic to the southeastern Qinghai–Tibet Plateau, offers a unique opportunity to examine these effects. The species was reassessed in 2024 as Least Concern (previously Near Threatened) [1,2]; its population trend is stable across a large and largely undisturbed range, although it still faces local habitat degradation and human disturbance [3]. Notably, the same assessment highlights that the species remains locally common around Tibetan monasteries where birds are fed supplementary barley, and suggests that this provisioning may bolster the population; the microbiological consequences of such provisioning, however, have not been examined.

Diet is a dominant force shaping gut microbial communities [4]. Captivity predictably alters diet, and captive birds of several species show reduced gut bacterial diversity relative to wild conspecifics [5,6,7]; in small passerines, gut microbial composition also varies with season [8]. A recent meta-analysis confirmed that captivity consistently shifts Firmicutes-to-Bacteroidetes ratios and reduces alpha diversity across avian taxa [9], and the translational potential of microbiome management for captive animal health has been recognized [10]. For phasianids specifically, Shen et al. [11] reported Proteobacteria as the dominant gut phylum. Pheasant gut microbiota also reflect host genetic variation [12], and, in mammals, Li [13] showed that captive golden snub-nosed monkeys carry significantly higher faecal loads of *Lactobacillus* (*p* < 0.05) and Bifidobacterium (*p* < 0.01) than wild individuals. However, most avian gut microbiota studies, including those of pheasants, have examined only bacteria [14,15]. Whether fungi—a substantial but understudied component of the gut ecosystem—respond similarly to environmental shifts is far less clear. Significant gut fungal differentiation between wild and captive animals has been reported in North Chinese leopards [16] and captive waterfowl [17]. Only a handful of studies have simultaneously profiled both kingdoms in birds [18,19]. To our knowledge, no dual-kingdom characterization has been reported for the genus *Crossoptilon*, and simultaneous bacterial and fungal profiling remains uncommon among phasianids generally. While the gut microbiota of the brown eared pheasant (*Crossoptilon* mantchuricum) has been examined using 16S rRNA sequencing in a captive seasonal study [20], a comprehensive bacterial-and-fungal assessment across wild and managed populations is lacking for the genus *Crossoptilon*.

A third living condition, rarely studied, is the monastery habitat [21]. At Xiongse Temple near Lhasa, Tibetan eared pheasants roam freely but receive supplementary highland barley from monks and visitors. We hypothesized that this condition—natural habitat plus anthropogenic food input—might support gut microbial communities distinct from both fully captive and fully wild birds. To test this, we collected fecal samples from birds at three sites spanning a captivity gradient and sequenced both the bacterial 16S ribosomal RNA gene (V3–V4 region) and the fungal internal transcribed spacer 1 (ITS1) region.

We acknowledge at the outset that this is an exploratory study. The fully captive group (Group A) comprised only three 16S samples; the birds at this site were not captive-bred but had been admitted to the Nyingchi Wildlife Rescue Station for rehabilitation following injury or debilitation, and because admissions of this protected species are rare and unpredictable, only a small number of rescued birds was available during the study period. For this reason, Group A is treated as a small, cautionary case rather than a well-replicated treatment group: the contrast between the captive and the two free-ranging groups underpins our main conclusion, but with *n* = 3 we report effect sizes alongside *p*-values throughout, and we used leave-one-out sensitivity analysis to check that no single captive sample drove the pattern (Supplementary Figure S1). The best-powered comparison available in this design is between the two free-ranging groups (B vs. C, *n* = 7 and 6), and we rely on it when asking whether human proximity per se, as opposed to captivity, is associated with microbiota change.

## 2. Materials and Methods

### 2.1. Sample Collection

Fresh fecal samples were collected in summer 2021 under three living conditions: (1) Fully captive (Group A, Nyingchi Wildlife Rescue Station, Xizang): wild-born birds admitted to the station for rescue and rehabilitation following injury or debilitation, where they were kept together as a group and maintained entirely on artificial feeding; 16S rRNA samples MJA1–MJA3 (*n* = 3), ITS samples MJA4–MJA6 (*n* = 3). (2) Wild (Group B, Biri Sacred Mountain, Nyingchi): free-foraging with no anthropogenic food supplementation; 16S rRNA samples MJB1–MJB7 (*n* = 7), ITS samples MJB8–MJB14 (*n* = 7). (3) Semi-wild monastery (Group C, Xiongse Temple, Lhasa): free-ranging in natural habitat with supplementary feeding (highland barley) from monks and visitors; 16S rRNA samples MJC1–MJC6 (*n* = 6), ITS samples MJC7–MJC11 (*n* = 5). Total: 16 bacterial samples and 15 fungal samples (31 in total). Donors were not individually identified: at each site, fresh pellets were collected at random from the ground surface, and each sample was assigned its own field identifier, so that no sample was used for both kingdoms. The three sites were the Nyingchi Wildlife Rescue Station (29.6525° N, 94.3812° E; 3213 m a.s.l.), Biri Sacred Mountain near Nyingchi (29.6196° N, 94.4296° E; 3689 m a.s.l.), and Xiongse Temple near Lhasa (29.4455° N, 91.0281° E; 4353 m a.s.l.). The two Nyingchi sites (Groups A and B) lie about 6 km apart, whereas Xiongse Temple (Group C) is about 325 km to the west, near Lhasa. Freshness was judged from the appearance of the material at the time of collection, which is an approximate criterion only; the exact interval since defecation, the time of day and the ambient temperature were not recorded. To limit contamination, samples were taken with sterile or single-use instruments; the outer surface of the pellet was scraped away and the inner material retained; gloves and instruments were changed between samples; and each sample was sealed into a sterile 5 mL cryovial immediately, frozen in liquid nitrogen and later transferred to −80 °C, so that it was not returned to the ground after collection. Contamination acquired at the ground surface therefore cannot be excluded, and this is of particular concern for the fungal (ITS1) data, which we treat as provisional (Section 4.5). Reference [22] is cited only as evidence that storage of up to 4 h after defecation, at known intervals, has little effect on overall α- and β-diversity; because that study examined samples of known exposure time, it does not establish that samples of unknown exposure time are unaffected, and we do not rely on it for that purpose. Antibiotic treatment history for the rescued birds was not systematically recorded, and we therefore cannot exclude recent antimicrobial exposure as a confounding factor (see Section 4.5).

### 2.2. DNA Extraction, PCR Amplification, and High-Throughput Sequencing

Total DNA was extracted using the TGuide S96 Magnetic Bead Soil/Stool DNA Extraction Kit (Tiangen Biotech, Beijing, China). The 16S rRNA V3–V4 region was amplified with primers 338F/806R, and the ITS1 region with ITS1F/ITS2. PCR products were purified and sequenced on the Illumina Novaseq 6000 platform (Biomarker Technologies, Beijing, China). The bacterial and fungal libraries were prepared and sequenced as two separate contracts; because every comparison reported here is made within a single kingdom, this potential batch effect does not confound any group contrast, although it precludes direct quantitative comparison of diversity between the two kingdoms.

### 2.3. Bioinformatics and Statistical Analysis

Raw reads were quality-filtered with Trimmomatic v0.33, primers were removed with cutadapt 1.9.1, and read pairs were merged on overlap with FLASH v1.2.7, as documented in the sequencing provider’s delivery report. The merged sequences were then clustered into operational taxonomic units (OTUs) at 97% similarity using USEARCH v10.0 [23] with chimera removal [24]. Singletons were removed before the OTU table was delivered: in the analysed tables no OTU has a total count of one or two reads across the libraries (16S, 916 OTUs; ITS, 1260 OTUs). We used 97%-similarity OTU clustering rather than an amplicon sequence variant (ASV) workflow, because the OTU table is the format in which the data were delivered and because it provides a widely used basis for cross-study comparison. We did not carry out an ASV-based re-analysis, and therefore make no claim that the two approaches would give equivalent results for this data set. Taxonomy was assigned against SILVA v132 for bacteria [25] and UNITE v8.0 for fungi [26], with a confidence threshold of 0.7 applied during classification. SILVA v132 is an older release (release 138.2 appeared in July 2024), and we did not test how sensitive these assignments are to the reference database version, so the phylum- and genus-level labels reported here should be read as assignments under v132. The sequencing provider calculated the abundance-based coverage estimator (ACE), Chao1, Simpson and Shannon indices on unrarefied data, and rarefaction curves were used to confirm adequate sequencing depth (see Results); of these, OTU richness, Chao1, Shannon and Simpson were analysed here. Shannon values are reported on a log base 2 scale, as produced by the analysis pipeline; multiplying by 0.693 gives the natural-logarithm values used in most published avian microbiome studies. Chloroplast and mitochondrial OTUs were removed before the taxonomic-composition analyses (909 of the 916 OTUs detected in this dataset retained, 91.2% of reads), because they represent plant and host organelle DNA rather than bacteria, whose 16S genes are also amplified by broad-range bacterial primers [27]; chloroplast sequences alone accounted for 0.07%, 8.10% and 14.11% of reads in Groups A, B and C, respectively. These sequences are reported separately as a qualitative index of plant-derived material (Supplementary Table S1), following the approach used to track dietary plant content along captivity gradients in primates [28], but the index is not quantitative, because chloroplast read recovery depends on chloroplast density in the ingested tissue and on DNA degradation, and it cannot resolve diet composition. The corresponding complete-table values are provided in Supplementary Table S2 for comparison. Principal coordinate analysis (PCoA) and permutational multivariate analysis of variance (PERMANOVA) were computed on Bray–Curtis and Binary Jaccard distances, which require no phylogenetic tree, on the organelle-free table, and on Weighted and Unweighted UniFrac distances, which do require a reference tree, on the complete OTU table; the UniFrac metrics could therefore not be recomputed after organelle removal. The tree underlying these distances is a Newick tree of the representative OTU sequences that accompanies the OTU table; it carries branch lengths and nodal support values and was used as supplied. UniFrac is independent of tree rooting, so no re-rooting was performed, and no sequences were removed from the tree beyond those already excluded from the OTU table, which in this case means that the organelle sequences were present in the tree for the UniFrac calculations. UniFrac was not applied to the ITS1 data, because alignment of ITS1 between phylogenetically distant fungal taxa is unreliable and no adequately supported fungal tree was available. For this reason, and so that the bacterial and fungal comparisons rest on the same kind of metric and on a single data basis, only the two non-phylogenetic metrics (Bray–Curtis and Binary Jaccard) were treated as primary and carried into Table 1; the Weighted and Unweighted UniFrac results are reported for reference only (PCoA in Supplementary Figure S2). These four matrices for the bacterial data (Bray–Curtis, Binary Jaccard, Weighted UniFrac and Unweighted UniFrac), together with the two fungal matrices, are included in the data deposit, so that every ordination and PERMANOVA result reported here can be reproduced exactly. To confirm that the results do not depend on sequencing depth, the organelle-free tables were rarefied, after organelle removal, to a common depth: 39,806 reads per sample for 16S, which is the smallest organelle-free bacterial library, and 67,334 for ITS. These rarefied tables were analysed in 100 independent draws for the α-diversity indices and 50 draws for PERMANOVA (999 permutations per draw; Supplementary Table S3). The unrarefied organelle-free analysis is the primary analysis throughout; the rarefied draws are stability checks, not additional biological replicates. Mean *p*-values across draws are tabulated for descriptive purposes only and are not used as a test statistic, because averaging permutation *p*-values does not constitute a validated statistical test; stability is judged instead by the proportion of draws in which a given result held. PERMANOVA used 9999 permutations. The number of distinct ways of partitioning the samples into the observed group sizes is 120 for Group A versus B (3 vs. 7), 84 for A versus C (3 vs. 6), 1716 for B versus C (7 vs. 6) and 960,960 for the three-group test in the bacterial data set, and 120, 56 and 792 for the three pairwise contrasts and 360,360 for the three-group test in the fungal data set. Because every pairwise partition space is smaller than 9999, the pairwise tests enumerate the complete space and their *p*-values can attain the exact minima 1/120 = 0.008, 1/84 = 0.012 and 1/1716 = 0.0006 (bacteria) and 1/120 = 0.008, 1/56 = 0.018 and 1/792 = 0.001 (fungi). The three-group tests, in contrast, draw 9999 permutations from a space of 960,960 (bacteria) or 360,360 (fungi) partitions, so their *p*-values are Monte Carlo estimates with a floor of 1/10,000 = 0.0001. To characterise dissimilarity independently of *p*-values, we also computed mean pairwise Bray–Curtis dissimilarities within and between groups, with 95% confidence intervals obtained by bootstrapping samples within groups (2000 resamples, percentile method); these distances were computed on the organelle-free table. Homogeneity of multivariate dispersion was assessed with betadisper, using distances to group centroids in principal-coordinates space, a one-way ANOVA on those distances and 9999 permutations. In the bacterial data, no heterogeneity was detected (Bray–Curtis *F* = 2.15, *p* = 0.153; Binary Jaccard *F* = 3.93, *p* = 0.050); in the fungal data, Bray–Curtis gave no evidence of heterogeneity (*F* = 0.66, *p* = 0.608), whereas Binary Jaccard did (*F* = 23.61, *p* = 0.0001). Inspection of the underlying distances shows that the latter reflects the very small spread among the three captive samples (distances to centroid 0.294–0.306, versus 0.328–0.358 in the wild and 0.331–0.354 in the monastery group) rather than a biologically meaningful difference in dispersion, and with only three captive samples the test is unreliable in either direction. No correction for unequal group sizes was applied because the test is permutation-based; the small and unequal group sizes are instead reflected in the caveat above. We therefore do not treat the non-significant results as demonstrating homogeneity of dispersion, nor as establishing that the PERMANOVA separations arise solely from differences in community location. Because Group A contained only three samples, the attainable *p*-values for comparisons involving this group are coarsely spaced, and these comparisons were therefore interpreted alongside effect sizes (*R*^2^) rather than as precise significance levels. No adjustment was made for multiple pairwise comparisons. In the bacterial data set, the significant pairwise *p*-values (0.008 and 0.012) lie below the Bonferroni threshold (α = 0.05/3 = 0.0167). In the fungal data set, the A versus C contrast cannot fall below 1/56 = 0.01786, which already exceeds this threshold, so that comparison cannot reach Bonferroni-adjusted significance at these sample sizes and its nominal *p*-value (0.018) is reported as exploratory; the A versus B contrast (*p* = 0.016, attainable minimum 0.008) lies at the edge of the threshold and is likewise interpreted conservatively.

Because Group A contained only three samples, normality and variance homogeneity cannot be assessed, and a small sample size is not in itself a reason to prefer a parametric test. We therefore define the Kruskal–Wallis test as the primary test for among-group differences in α diversity, with pairwise two-sided Mann–Whitney U tests performed where the omnibus test was significant; one-way analysis of variance (ANOVA) with Tukey HSD is reported alongside it as a secondary, parametric description. Agreement between the two approaches is reported but is not treated as validation of either, and where they disagree the non-parametric result is used and the disagreement is stated explicitly. All α- and β-diversity analyses were computed on the organelle-free bacterial table, so that a single data basis is used throughout, and the four α-diversity indices analysed here (OTU richness, Chao1, Shannon and Simpson) are reported in full in Supplementary Table S4. No correction for multiple testing was applied across these four indices, because they quantify different and non-interchangeable aspects of diversity; we note, however, that the bacterial OTU-richness comparison (*p* = 0.030) would not reach the Bonferroni threshold for four indices (0.0125), and we therefore describe that difference as consistent across tests rather than as corrected-significant. Taxa enriched in each group were identified by linear discriminant analysis effect size (LEfSe), using the sequencing provider’s ‘LDA4 less-strict’ analysis (*p* < 0.05) on the complete OTU table. In SILVA v132, chloroplast sequences are classified within the phylum Cyanobacteria, so the Chloroplast and Cyanobacteria entries in the bacterial LEfSe output (Section 3.3, Supplementary Figure S3) are not independent findings: chloroplast reads accounted for 0.07%, 8.10% and 14.11% of reads in Groups A, B and C, and made up the great majority of the reads assigned to Cyanobacteria in those groups (0.08%, 8.46% and 14.84%, respectively). We therefore treat this signal as plant-derived rather than as evidence of cyanobacterial colonisation, and after removal of organelle sequences the residual non-chloroplast Cyanobacteria accounted for only 0.01%, 0.43% and 0.83% of the organelle-free reads (phylum-level values in Section 3.3). Cyanobacteria are not in principle identifiable with plant material, and this interpretation is restricted to the present data set. For ITS, the original Biomarker LEfSe output was limited to a two-group (MJA vs. MJB) comparison; we therefore carried out a three-group differential-abundance analysis directly on the BIOM-formatted OTU table, using non-parametric Kruskal–Wallis tests followed by pairwise Mann–Whitney U tests (*p* < 0.05), and ranked the resulting taxa by their mean relative abundance in the group in which they were enriched; this ranking is descriptive only and is not a measure of effect size. All differential-abundance results (LEfSe and the non-parametric tests reported below) are treated as exploratory rather than confirmatory: with only three captive-group samples, a two-sided Mann–Whitney test cannot return a *p*-value below 0.0167 for Group A versus Group B (*n* = 3 vs. 7) or 0.0238 for Group A versus Group C (*n* = 3 vs. 6), and Benjamini–Hochberg correction across the 1203 genus-level pairwise comparisons yielded no taxon with q < 0.05 (minimum q = 0.16); this reflects the size of the comparison family together with the small group sizes, but it does not establish that FDR-controlled detection is impossible at this sample size. We therefore do not use these analyses to support any of our conclusions, and present them as hypothesis-generating only, consistent with the documented instability of differential-abundance tools in small datasets [29]. Analyses were conducted with Python 3.13 (NumPy 2.5.0, SciPy 1.18.0, Matplotlib 3.11.0, NetworkX 3.6.1) together with the sequencing provider’s standard pipeline.

### 2.4. Ethical Statement

The animal study protocol was approved by the Institutional Review Board of Institute of Plateau Biology of Xizang Autonomous Region (approval number: LL2201035; date of approval: 5 April 2021).

## 3. Results

### 3.1. Sequencing Data Overview

The 16 bacterial samples yielded 1,207,222 valid sequences (mean 75,451 per sample; average read length 415–428 bp); of these, 965,833 (80.0%) were retained in the final OTU table after quality filtering, chimera removal and clustering at 97% similarity. The 15 fungal samples yielded 1,179,393 valid sequences (mean 78,626 per sample; average read length 242–256 bp). Per-sample sequencing statistics are provided in Supplementary Table S5. Rarefaction curves approached plateaus and Good’s coverage exceeded 0.99 in all samples, confirming sufficient sequencing depth (Supplementary Figure S4). Figure 1 summarizes the bacterial and fungal responses across the three groups.

### 3.2. Bacterial Community Alpha Diversity

After clustering at 97% similarity, 916 bacterial OTUs were obtained across all 16 samples, with 462 OTUs shared across all three groups (Supplementary Figure S5; detailed data in Supplementary Table S2).

All α-diversity values are computed on the organelle-free bacterial table (Supplementary Table S4). Group A had the lowest observed Shannon diversity of the three groups (2.93, log2 basis; Figure 2), but neither test detected an among-group difference (Kruskal–Wallis *p* = 0.147; ANOVA *F* = 2.22, *p* = 0.148), consistent with the limited power imposed by the small Group A sample size (*n* = 3). OTU richness, by contrast, differed among groups under both tests (Kruskal–Wallis *H* = 7.01, *p* = 0.030; ANOVA *F* = 7.49, *p* = 0.007), with Group A the lowest (272.3 ± 13.9 versus 554.6 ± 55.6 and 602.5 ± 48.8, mean ± SE), and Chao1 showed the same direction and was likewise significant under both tests (Kruskal–Wallis *H* = 7.42, *p* = 0.025; ANOVA *F* = 4.70, *p* = 0.029). Simpson did not differ among groups (Kruskal–Wallis *p* = 0.487; ANOVA *p* = 0.683), so we draw no conclusion from it. Computing these indices on the complete table instead of the organelle-free table changed the Shannon values only slightly (2.94, 5.39 and 5.75) and did not alter any of these conclusions; the rarefied analysis gave the same pattern (Supplementary Table S3).

### 3.3. Bacterial Community Composition and Differential Taxa

At the phylum level (Figure 3), Firmicutes was dominant across groups but substantially higher in Group A (75.91%) than Groups B (50.78%) and C (65.82%). Bacteroidetes, Actinobacteria, Acidobacteria, and Verrucomicrobia were all markedly less abundant in Group A (detailed data in Supplementary Table S6). Correspondingly, the Firmicutes-to-Bacteroidetes ratio was far higher in Group A (359.8, computed as the mean of per-sample ratios) than in Groups B (15.7) and C (12.6), consistent with the captivity-associated shift in this ratio reported across avian taxa [9]. Chloroplast and mitochondrial OTUs were excluded from these composition estimates; plant-derived chloroplast sequences accounted for 0.07%, 8.10% and 14.11% of reads in Groups A, B and C, respectively, and are reported separately (Supplementary Table S1).

At the genus level (Figure 4), Group A was overwhelmingly dominated by *Lactobacillus* (51.4%), followed by *Escherichia–Shigella* (21.2%); note, however, that *Escherichia–Shigella* was also abundant in the wild group (13.6%) and that the Group A versus B difference for this genus was not statistically supported (Section 4.2). Groups B and C had more balanced communities, with *Staphylococcus* (11.3% and 16.4%), Enterococcus (13.7% and 2.4%), Ruminococcaceae_UCG-014 (4.8% and 4.7%) and uncultured Muribaculaceae (2.4% and 3.5%) among the major constituents, and Lysinibacillus reaching 6.9% in Group C (detailed data in Supplementary Table S7).

LEfSe (Biomarker ‘LDA4 less-strict’ analysis; *p* < 0.05) identified group-specific enriched taxa (Figure 5, Supplementary Figure S3): Group A—Lactobacillaceae/*Lactobacillus* (LDA = 5.71) and Peptostreptococcaceae/*Romboutsia* (LDA = 4.72); Group B—Rhizobiales (LDA = 4.51) and *Ruminococcaceae_UCG-014* (LDA = 4.62); Group C—*Staphylococcus* (LDA = 5.11), chloroplast (LDA = 5.15), *Lysinibacillus* (LDA = 4.81), and Bacteroidales (LDA = 4.85). Repeating these comparisons with two-sided Mann–Whitney tests on the same table gave enrichment of *Romboutsia* in Group A (*p* = 0.017 vs. Group B; *p* = 0.024 vs. Group C) and lower abundance of Verrucomicrobiae and Actinobacteria in Group A than in either free-ranging group (*p* = 0.017 and 0.024, respectively). These values are the smallest attainable at these sample sizes, and none survive Benjamini–Hochberg correction at the scale of the genus-level testing performed here (1203 comparisons; minimum q = 0.16). All differential-abundance results are therefore reported as exploratory.

To ask whether the groups differed in which taxa were present, and not only in their relative proportions, we defined the core community of each group as the genera detected in at least 80% of its samples; because the groups differ in size, this threshold corresponds in practice to all three Group A samples, at least six of the seven Group B samples and at least five of the six Group C samples, so the criterion is slightly stricter for Group A and the contraction reported below is, if anything, conservative. Core richness increased progressively from Group A (91 genera) through Group B (181) to Group C (251); 77 genera were core to all three groups, and 154 genera were detected in at least 80% of all 16 samples. The principal genera were present in every sample irrespective of group: *Lactobacillus*, *Escherichia–Shigella*, *Romboutsia*, *Staphylococcus* and *Enterococcus* each occurred in 100% of samples in all three groups. The captive group therefore shows a contraction in the number of prevalent genera—its core was roughly half the size of the wild group’s—together with a redistribution of abundance among the genera that persist: *Lactobacillus* was detected in every sample, yet rose from 8.2% (Group B) and 8.4% (Group C) to 51.4% in Group A.

### 3.4. Bacterial Community Beta Diversity and PERMANOVA

PCoA based on Bray–Curtis distance showed clear separation of Group A from Groups B and C (Figure 6). PERMANOVA confirmed significant among-group differentiation for both non-phylogenetic metrics on the organelle-free table (Table 1): Bray–Curtis *R*^2^ = 0.309 (*F* = 2.908, *p* = 0.001) and Binary Jaccard *R*^2^ = 0.309 (*F* = 2.905, *p* < 0.001). Because these two metrics require no phylogenetic tree, they could be recomputed after organelle removal and are treated as the primary β-diversity metrics. Weighted and Unweighted UniFrac, which do require a reference tree, gave a consistent pattern on the complete OTU table (*R*^2^ = 0.379, *p* = 0.004 and *R*^2^ = 0.323, *p* = 0.002, respectively) but are reported for reference only; the bacterial and fungal comparisons therefore rest on the same kind of metric and on a single data basis. The organelle-free pairwise contrasts were *R*^2^ = 0.336 (*p* = 0.008) for A vs. B, 0.407 (*p* = 0.012) for A vs. C and 0.083 (*p* = 0.423) for B vs. C under Bray–Curtis, and 0.292 (*p* = 0.008), 0.386 (*p* = 0.012) and 0.103 (*p* = 0.158) under Binary Jaccard. For the three-group test, in which 9999 permutations sample a space of 960,960 distinct partitions, the minimum achievable *p*-value was 0.0001; for the two pairwise contrasts involving Group A, the reported values (*p* = 0.008 and *p* = 0.012) are themselves the smallest attainable, corresponding to 1/120 and 1/84. On this organelle-free table, the mean Bray–Curtis dissimilarity between the monastery and wild groups was 0.676 (95% bootstrap CI 0.562–0.782), a value that falls between the mean within-group dissimilarities of the monastery (0.674) and wild (0.684) samples, whereas both contrasts involving the captive group were substantially higher (A vs. B 0.846, 95% CI 0.754–0.893; A vs. C 0.884, 95% CI 0.861–0.913). The PERMANOVA results were also stable under rarefaction, assessed as a stability check rather than as a replicate analysis: across 50 independently rarefied datasets, the three-group model gave a mean *R*^2^ of 0.309, A vs. B and A vs. C were significant in all 50 rarefied datasets, and B vs. C was non-significant in all 50 (Supplementary Table S3).

Pairwise PERMANOVA (Table 1) revealed that Group A differed significantly from both Groups B (*R*^2^ = 0.292–0.336, *p* = 0.008) and C (*R*^2^ = 0.386–0.407, *p* = 0.012). Groups B and C did not differ significantly under either metric (*R*^2^ = 0.083–0.103, *p* = 0.158–0.423), indicating that community divergence was most strongly associated with captivity rather than with human proximity per se; because captivity co-varied with site elevation and vegetation in our design, however, this association should not be read as a purely causal effect of captivity (see Limitations). We note that non-significant PERMANOVA does not prove equivalence, especially with modest sample sizes; the low *R*^2^ values (all < 0.13) are consistent with, but do not establish, a biologically meaningful similarity. Weighted UniFrac PCoA supported this pattern (Supplementary Figure S2).

### 3.5. Fungal Community Alpha Diversity

Across the 1260 fungal OTUs, the mean number of OTUs detected per sample did not differ significantly among groups (A: 558.3 ± 9.1; B: 584.4 ± 26.2; C: 564.2 ± 9.5, mean ± SE; *F* = 0.38, *p* = 0.695; Supplementary Table S8). A total of 720 OTUs were shared across all three groups (Supplementary Figure S6). Shannon diversity ranged from 7.17 to 7.40 on a log2 basis (4.97–5.13 as natural logarithms), reflecting the intrinsically greater taxonomic richness of fungal communities profiled by ITS1, and varied far less among groups than bacterial Shannon (2.93–5.79 log2; detailed data in Supplementary Table S8). Under the parametric test, the among-group difference in fungal Shannon was significant, and to a nearly identical degree, in both the unrarefied and the rarefied data (*F* = 6.42 and 6.39, respectively; *p* = 0.013 in both cases; Supplementary Table S3); the Kruskal–Wallis test on the same data, however, did not reach significance (*H* = 5.82, *p* = 0.054). Because the non-parametric test is the primary one here, we report this difference as test-dependent and do not interpret it as an established among-group difference; the effect is in any case small relative to that in bacteria. Fungal richness and Chao1 did not differ among groups under either test (all *p* ≥ 0.111).

### 3.6. Fungal Community Composition and PERMANOVA

At the phylum level (Figure 7), Ascomycota (79.4–80.9%) and Basidiomycota (9.4–10.2%) dominated consistently across groups (detailed data in Supplementary Table S9).

At the genus level, *Fusarium* (5.5–6.2%), *Purpureocillium* (5.0–5.5%), and *Aspergillus* (5.0–6.7%) predominated, with no significant differences among groups (Supplementary Figure S7).

PERMANOVA showed a weaker compositional signal for the fungal communities than for the bacterial ones (Table 2): Bray–Curtis *R*^2^ was 0.194 (*p* = 0.008), lower than the bacterial value of 0.309, while Binary Jaccard was non-significant (*R*^2^ = 0.144, *p* = 0.447). Among pairwise comparisons under Bray–Curtis, A vs. B (*R*^2^ = 0.185, *p* = 0.016) and A vs. C (*R*^2^ = 0.250, *p* = 0.018) were nominally significant, whereas B vs. C was not (*p* = 0.943). Both values lie at or near the smallest attainable at these group sizes (1/120 and 1/56, respectively); after Bonferroni correction (α = 0.0167) the A versus C contrast cannot remain significant, since its attainable minimum (0.01786) already exceeds α, and both comparisons are therefore interpreted as exploratory. No pairwise comparison was significant under Binary Jaccard.

The three-group differential-abundance analysis of the ITS data (see Methods) identified distinct taxa enriched in each group (Supplementary Figure S8): Group A was characterized by Saccharomycetales, Debaryomycetaceae, *Debaryomyces*, and Thelephoraceae; Group B by Eurotiales, Aspergillaceae, and Phaffomycetaceae; and Group C by Saccharomycetaceae, *Saccharomyces*, and *Leohumicola*. *Aspergillus* abundance was higher in Group B (6.7%) than Group A (5.0%), suggesting it is an intrinsic core community member rather than a captivity-introduced risk factor.

### 3.7. Bacterial–Fungal Differential Response Pattern

The bacterial and fungal responses diverged sharply (Figure 1). Bacterial Shannon varied much more across groups (2.93–5.79 on a log2 basis; 2.03–4.01 as natural logarithms) than fungal Shannon (7.17–7.40 log2; 4.97–5.13 ln). Bacterial Bray–Curtis PERMANOVA *R*^2^ (0.309) was about 1.6 times the fungal value (0.194), and Binary Jaccard was significant for bacteria (*R*^2^ = 0.309, *p* < 0.001) but not for fungi (*p* = 0.447). Bacterial genus richness, calculated after removal of chloroplast and mitochondrial OTUs, was approximately two-thirds as high in Group A (263 genera) as in Groups B (389) and C (381); fungal genus richness varied by 23% across groups (A: 257, B: 317, C: 291), a narrower range than the 48% observed in bacteria. At the phylum level, Firmicutes fell from 75.9% (A) to 50.8% (B), while Ascomycota held steady at 79–81%. The overall pattern is one of a stronger bacterial than fungal compositional signal, although the two *R*^2^ values are not strictly comparable, being derived from datasets that differ in OTU number, marker gene and sequencing depth, and they should not be read as a measure of intrinsic sensitivity.

## 4. Discussion

We compared the gut microbiota of the Tibetan eared pheasant across three living conditions that represent increasing degrees of human management: full captivity at a rescue station, free-ranging with human provisioning at a monastery, and the wild. Two patterns emerged. The primary one concerns management: the captive group showed a wholesale restructuring of the bacterial community, whereas we did not detect a significant difference between the monastery birds and wild birds. Superimposed on this was a secondary, kingdom-level pattern: the compositional signal detected for fungal communities was far weaker than that for bacteria; whether this reflects a genuine difference in the sensitivity of the two kingdoms cannot be resolved here, because the two datasets differ in marker, OTU number and sequencing depth.

### 4.1. High Gut Bacterial Diversity in the Monastery Population

We had expected the monastery group to fall somewhere between captive and wild. It did not: Group C Shannon diversity (5.79) slightly exceeded even the wild group (5.54), and its community structure did not differ significantly from that of wild birds (Table 1). This result challenges the assumption that any anthropogenic food input degrades wildlife microbiota.

What might explain it? The elevated Chloroplast sequences in Group C (14.1%, LDA = 5.15) point to plant material from supplementary feeding, primarily highland barley, as a likely contributor. Knutie et al. [30] showed that human food supplementation in Darwin’s finches can widen trophic niches and increase gut microbial diversity, and our data suggest a similar dynamic may operate at Xiongse Temple. The monastery effectively adds a dietary subsidy without removing natural foraging opportunities. In the primate captivity gradient in which this chloroplast index was first applied, semi-captive animals had lower plant-derived sequence content than wild ones [28]; here the pattern is reversed, with the provisioned monastery group exceeding the wild group, which is consistent with provisioning adding plant material rather than replacing it.

For conservation management, this is the most actionable result of the study. It implies that ex situ strategies need not be all-or-nothing: rather than removing birds from their habitat, management that retains natural foraging while allowing supplementary feeding, as practised at Xiongse Temple, was associated with a gut bacterial community in which we did not detect a significant difference from that of wild birds and with the numerically highest Shannon diversity of the three groups, although this difference was not supported by the omnibus test. This is consistent with experimental evidence that continuing to supply natural foods to captive animals preserves most of their native gut microbiota, whereas a switch to commercial rations causes substantial loss [31]. Applied to the Tibetan eared pheasant, these observations are consistent with prioritizing in situ protection and light-touch provisioning over transfer to full captivity, but do not establish that this is the better option, and suggest that temple-associated populations, already a widespread feature of this species’ range, may be better regarded as a conservation asset than as a disturbance. We caution, however, that our monastery sample derives from a single site; that the amount and composition of the supplementary barley were not quantified; that habitat, elevation and diet could not be separated in this design; that no host health, body condition or fitness was measured, so that no conservation or health benefit can be inferred from microbiota similarity alone; that these taxonomic data cannot establish causation; and that whether the pattern holds at other temples or under different feeding regimes remains to be tested in replicated, multi-site studies.

### 4.2. Captivity as an Extreme Perturbation of Gut Bacterial Communities

The captive birds (Group A, 16S *n* = 3) showed a dramatically simplified gut microbiota. Shannon diversity was the lowest of the three groups (2.93 on a log2 basis, versus 5.54 and 5.79; Figure 2), although the corresponding omnibus test was not significant, whereas OTU richness was significantly lower in Group A than in both free-ranging groups (Supplementary Tables S4 and S3). A single genus, *Lactobacillus*, accounted for over half of all sequence reads (51.4%; Figure 4). This degree of dominance is striking and suggests an ecosystem-level perturbation. Metagenomic profiling of ring-necked pheasants similarly documented Firmicutes dominance and *Lactobacillus* enrichment [15]. Phasianid microbiota studies have similarly reported captive diet effects on community structure [11,12,20,32]. This pattern in *C. harmani* extends earlier findings from the closely related brown eared pheasant (*C. mantchuricum*), where Firmicutes-dominated communities shifted seasonally but remained within the range of natural variation [20], whereas our captive group reached a markedly distinct state of near-complete *Lactobacillus* dominance. The host is a high-elevation, primarily plant-foraging Galliform whose natural diet of roots, leaves, tubers, seeds and fruits (supplemented by insects during the breeding season) shapes a gut community adapted to fibrous, carbohydrate-rich forage [3,4]; captivity replaces this diet with an artificial ration of unrecorded formulation, which may plausibly favour the rapid-growing lactic acid bacteria that dominate Group A [9].

The PERMANOVA results support this interpretation. Under both non-phylogenetic metrics, Group A differed from Groups B and C with *R*^2^ values ranging from 0.292 to 0.407 (*p* ≤ 0.012; Table 1). Groups B and C did not differ significantly under either metric (*R*^2^ = 0.083–0.103, all *p* > 0.05). This pattern suggests that the divergence was associated primarily with captivity, with its attendant dietary simplification and spatial confinement, rather than with human proximity per se, although site elevation and vegetation co-vary with captivity in our design (see Limitations).

*Lactobacillus* blooms under captive conditions are well-documented. Species identity and dietary composition both influence gut microbiota structure in captive pheasants [32]. Li [13] reported similar enrichment in captive golden snub-nosed monkeys, and Fu [33] found elevated *Enterococcus* in captive greylag geese relative to sympatric wild cranes. Xiao et al. [34] demonstrated experimentally that native starch in captive diets directly increases *Lactobacillus* abundance across nonpasserine birds. Where captive diets are rich in simple carbohydrates, rapid-growing lactic acid bacteria are favoured [35]; whether this applies to the ration used here could not be determined, because its composition was not recorded. The concurrent enrichment of Peptostreptococcaceae (Romboutsia; Figure 5; 5.20% in Group A versus 0.16% in both free-ranging groups) is noteworthy, although taxonomic data alone cannot show whether it contributed to the observed community simplification. Two non-exclusive explanations have been proposed for this genus in birds: it has been linked to high-protein, animal-based diets in raptors [36], and it is enriched in captive cranes maintained on plant-based rations [37]; it is also a butyrate-producing genus implicated in immune homeostasis [38]. Because the captive ration here was a uniform artificial feed of unrecorded composition, we cannot separate a dietary-protein effect from a general effect of captivity, and we flag both as plausible. *Escherichia–Shigella* was numerically highest in Group A (21.25%), but this difference was not statistically supported (Mann–Whitney *p* = 0.83 vs. Group B; *p* = 0.55 vs. Group C), it was driven by single samples in both the captive and the wild group (one wild-group sample reached 82.2%, while the third captive-group sample had 0.15%), and a recent comparison of captive and wild Siberian cranes found this genus to be more abundant in wild birds and in soil, interpreting it as a marker of environmental exposure rather than of disease risk [37]. Our data and that study therefore argue against treating *Escherichia–Shigella* abundance as a necessary consequence of captive housing; it appears instead to track environmental and dietary exposure, which differed among all three groups, and enteric taxa of this kind can also vary with host health status in captive wildlife [39], which we did not assess.

### 4.3. A Weaker Fungal Compositional Signal

In contrast to the bacterial response, the compositional signal detected for fungal communities was weaker. Bray–Curtis PERMANOVA was significant (*R*^2^ = 0.194, *p* = 0.008; Table 2), whereas Binary Jaccard was entirely non-significant (*p* = 0.447). This combination is consistent with differentiation arising mainly from shifts in relative abundance rather than from wholesale compositional turnover: Bray–Curtis was significant for both contrasts involving Group A, whereas Binary Jaccard was non-significant for every pairwise comparison. Because the two metrics differ in whether they weight abundance, this suggests a contribution of relative abundance, but it does not establish the mechanism of differentiation. *R*^2^ values obtained from different marker sets, different sample sets and different numbers of OTUs are not comparable, and they cannot be read as evidence that fungal communities are biologically less sensitive than bacterial ones. San Juan et al. [18] reported a qualitatively similar finding in Brown Kiwi, in that captivity shifted both bacterial and fungal communities, although with different magnitudes, and Madden et al. [19] found that chronic stress and captivity altered both kingdoms in house sparrows, with bacterial communities showing greater divergence.

The strongest signal was a roughly two-fold enrichment of Saccharomycetes in Group A (16.9%) relative to Groups B (8.0%) and C (8.5%), consistent with fermentable carbohydrate substrates favoring yeast growth. *Aspergillus* was most abundant in the wild group (6.7% in Group B vs. 5.0% in Group A), arguing against the assumption that captivity universally concentrates fungal opportunists. Without species-level resolution we cannot exclude the possibility that pathogenic *Aspergillus* species (e.g., *A. fumigatus*) behave differently from saprotrophic congeners, but the overall pattern suggests *Aspergillus* is a widespread component of the gut mycobiome in this species.

Why fungal communities respond more weakly than bacteria to the same environmental gradient is an open question. One possibility is that the slower growth of eukaryotes and the distinct immune surveillance to which commensal fungi are subject buffer fungal communities against short-term dietary shifts [40]. Technical factors may also attenuate the detectable fungal signal: low fungal biomass in the gut, variable ITS copy number, length and sequence heterogeneity of ITS amplicons, and incomplete fungal reference databases can all compress apparent differentiation; thus the weaker fungal response should not be over-read as community stasis. Whatever the mechanism, the practical implication is that fungal profiles carried less information about living condition in this data set than bacterial profiles did; whether they could serve as stable markers for population monitoring remains untested, because we did not assess host specificity. Recent dual-kingdom profiling in migratory cranes [41] further supports the need to simultaneously assess bacteria and fungi to understand gut microbial responses to environmental change.

### 4.4. Comparison with Existing Literature

Recent studies employing simultaneous 16S rRNA and ITS profiling in birds have expanded our understanding of gut microbial ecology, but few directly compare the differential responses of bacteria and fungi to varying degrees of anthropogenic influence. San Juan et al. reported that captivity reduced both bacterial and fungal diversity in brown kiwi [18]; that both kingdoms responded is consistent with our observation, whereas the strength of their fungal response contrasts with the weaker fungal differentiation seen here. This discrepancy may reflect differences in captive management (intensive zoo housing versus semi-natural rescue station), or the greater phylogenetic distance between kiwi (Palaeognathae) and pheasants (Neoaves), given that host evolutionary history strongly constrains gut microbiota composition. Li et al. similarly observed no significant fungal diversity difference between two crane species despite marked bacterial community divergence [41], supporting the view that fungal communities may be more host-conserved across avian taxa; that comparison, however, was between host species, whereas ours is between management conditions within a single species, so the parallel is suggestive rather than direct. At the same time, Rowe et al. demonstrated that fungal communities in captive kiwi chicks were “more transitory” following dietary soil amendment [42], suggesting that the stability of gut fungal communities is not absolute but context-dependent: highly stable under dietary or habitat-level perturbation, but responsive to drastic changes in environmental microbial exposure. In the broader conservation context, Zhang et al.’s multi-omic study of golden snub-nosed monkeys [43] established that food provisioning occupies an intermediate position between wild foraging and full captivity in terms of gut microbial impact, a finding that closely mirrors our observation that the monastery group (provisioned birds in a semi-wild habitat) showed the numerically highest bacterial Shannon diversity. Taken together, these cross-taxon comparisons suggest that the pattern documented here—a stronger bacterial than fungal compositional signal—may represent a generalizable phenomenon in vertebrate gut ecosystems, albeit one whose expression is modulated by host phylogeny, perturbation type, and management intensity. Future studies integrating metagenomic or metabolomic approaches with 16S+ITS profiling across broader taxonomic sampling would help determine the mechanistic basis and phylogenetic generality of this divergent response pattern.

A recurring theme in vertebrate gut ecology is that diet and feeding habitat are among the strongest determinants of gut microbial composition, often rivalling or exceeding host phylogeny. In a recent multispecies synthesis of 60 wild mammals, feeding habit and host phylogeny jointly structured gut microbiota, with herbivores showing the highest alpha diversity and diet-related differentiation comparable to phylogenetic effects [44]. Across birds as a whole, however, gut microbiomes correlate only weakly with diet and host phylogeny [45], so the dietary signal documented here most likely reflects within-system dietary change (the shift to a uniform ration) rather than dietary category per se. The three groups compared here differ in exactly these respects: fully captive birds depend on a uniform artificial ration, wild birds forage on natural high-elevation vegetation, and monastery birds combine natural foraging with a barley subsidy. The elevated chloroplast sequences and the distinct bacterial composition of the provisioned monastery group, together with the contrasting, *Lactobacillus*-dominated community of the captive group, are therefore consistent with dietary differences contributing substantially to the patterns observed, rather than with ‘living condition’ acting as an abstract variable. Because diet, habitat, elevation and management could not be experimentally separated in our design, however, we interpret these patterns as consistent with, rather than diagnostic of, a dietary effect.

### 4.5. Limitations

Several limitations should be considered when interpreting these results. Most critically, Group A comprised only three 16S samples and three ITS samples; because samples were not linked to identified donors, the 16S and ITS datasets cannot be matched to individuals, which precludes direct per-individual bacterial–fungal correlation analysis. We used Kruskal–Wallis tests and permutation-based PERMANOVA to partially address this, but the small n means that within-group variance compromises statistical power. Our study is also cross-sectional (summer 2021 only), so seasonal dynamics are unknown. In addition, each management regime was represented by a single site, so management is fully confounded with location, and because samples were not attributed to identified birds, the samples within a site cannot be confirmed to be independent replicates of that regime; the conclusions drawn here therefore pertain to the three populations sampled rather than to captivity or to supplementary feeding in general. The captive birds had been admitted to the rescue station after injury or debilitation, so their clinical condition at admission, the treatment they received and their convalescence represent further differences from the two free-ranging groups and an alternative explanation for their distinct microbiota that our design cannot exclude. We did not record individual-level metadata (age, sex, body mass, body condition score), antibiotic treatment history for the rescued birds, or any host physiological or clinical health measures, so we draw no conclusions about host health, and the three sites differed systematically in altitude (Nyingchi Rescue Station 3213 m, Biri Sacred Mountain 3689 m, Xiongse Temple 4353 m) and vegetation type, meaning that captivity is confounded with elevation and vegetation in our design; consequently, the group differences we report cannot be attributed to captivity per se and should be interpreted as associations rather than causal effects. We quantified this confounding: because each site has a single elevation, elevation is a linear function of group membership, so a design containing both terms is rank-deficient and no partial analysis can separate them. A model using elevation as a linear predictor explained less of the community variation than the grouping factor (*R*^2^ = 0.179, adjusted *R*^2^ = 0.121, versus *R*^2^ = 0.309, adjusted *R*^2^ = 0.203), and adding elevation to the group model contributed no additional explained variance (*R*^2^ = 0.309; Supplementary Table S3). Moreover, the two free-ranging groups, which differ in elevation by 664 m, showed no detectable community difference (*R*^2^ = 0.083, *p* = 0.423; Supplementary Table S10), whereas the comparison spanning only 476 m of elevation (Groups A and B) differed strongly (*R*^2^ = 0.336, *p* = 0.008; Supplementary Table S10); the community pattern is therefore not a simple monotone function of elevation, although elevation cannot be ruled out as a contributing factor. In addition, this species is highly social, and social rank can shape food access, physiological stress, and gut microbial composition in group-living vertebrates [46]; because individual social rank was not recorded, it represents a further unmeasured source of within-group variation, particularly in the captive and provisioned groups. An exploratory genus-level co-occurrence network is provided as Supplementary Figure S9; because of the limited sample size and the compositional nature of relative-abundance data, its associations should not be interpreted as evidence of interspecies interactions. Diet was not measured in any group, and although plant-derived chloroplast sequences indicate greater plant intake in Groups B and C than in Group A, this index is not quantitative and is effectively collinear with group membership (it was near zero in every Group A sample); dietary effects therefore cannot be separated from site, elevation or season in this design. The formulation of the artificial ration fed to the captive birds and the duration of their captivity were not recorded, so the dietary explanation offered in Section 4.2 remains a hypothesis rather than a demonstrated mechanism. No extraction or PCR-negative controls were processed, and fungal (ITS) reads are especially prone to environmental contamination; because the monastery birds feed on provisioned barley in a human-modified setting, part of the fungal signal in Group C could in principle derive from environmental rather than gut sources, and the fungal results should therefore be regarded as provisional. The differential-abundance results should likewise be read as exploratory: after Benjamini–Hochberg correction across the 1203 genus-level comparisons, no taxon reached q < 0.05, so the enriched taxa reported above are candidates for future testing rather than established differences; this outcome reflects our comparison family and sample sizes, and should not be read as evidence that FDR-controlled detection is impossible in this system. Finally, amplicon sequencing limits us to taxonomic inference; functional inference would require shotgun metagenomics, metatranscriptomics or metabolomics, which were not part of this study.

Future work should expand the captive sample size, add longitudinal sampling across seasons, incorporate host physiological data, and conduct multi-site monastery sampling to test the generality of the Xiongse Temple pattern. Reassuringly, leave-one-out sensitivity analysis on the organelle-free table confirmed that removing any single Group A sample did not alter the overall pattern: the group mean Shannon diversity of Group A stayed between 2.8 and 3.0, against 5.5–5.8 for Groups B and C, and the three-group PERMANOVA remained significant in all three iterations (*p* = 0.004–0.011, *R*^2^ = 0.26–0.28; Supplementary Figure S1).

## 5. Conclusions

To our knowledge, no characterization of the gut microbiota of this species has been published, and this study is therefore the first to describe both its bacterial and its fungal gut communities and to compare them across living conditions that differ in the degree of human management. Because samples were not linked to identified donors, the two kingdoms are characterized at group level only, and no per-individual integration of the two communities, or of their relationship to one another, is possible. The captive group showed markedly lower bacterial OTU richness and a *Lactobacillus*-dominated community, while its Shannon diversity was also the lowest of the three groups although the corresponding omnibus test was not significant; the fungal compositional signal was weaker, and the metrics available suggest that it arose mainly from shifts in relative abundance rather than from turnover in composition (Section 4.3). Most importantly for conservation, the monastery condition (natural habitat with supplementary feeding of unquantified amount) was associated with a bacterial community in which we did not detect a significant difference from the wild and with the numerically highest Shannon diversity of the three groups (a difference not supported by the omnibus test). This is consistent with, but does not demonstrate, the hypothesis that light-touch, in-situ-oriented management is associated with a gut bacterial community whose dissimilarity from that of wild birds is no greater than that among wild birds themselves and smaller than that of fully captive birds; testing this hypothesis would require replicated, multi-site studies with direct measures of diet, host condition and fitness. Because living conditions co-varied with site elevation and vegetation, and because each condition was represented by a single site, these patterns should be read as associations that pertain to the three populations studied and warrant causal testing in controlled, replicated settings. These findings provide a preliminary, population-specific microbiological reference for the three populations sampled and a starting point for future validation; given the small sample sizes, the single sampling season and the absence of site replication, they do not establish a health-associated reference profile, nor thresholds that could be applied directly to management.

## Figures and Tables

**Figure 1:**
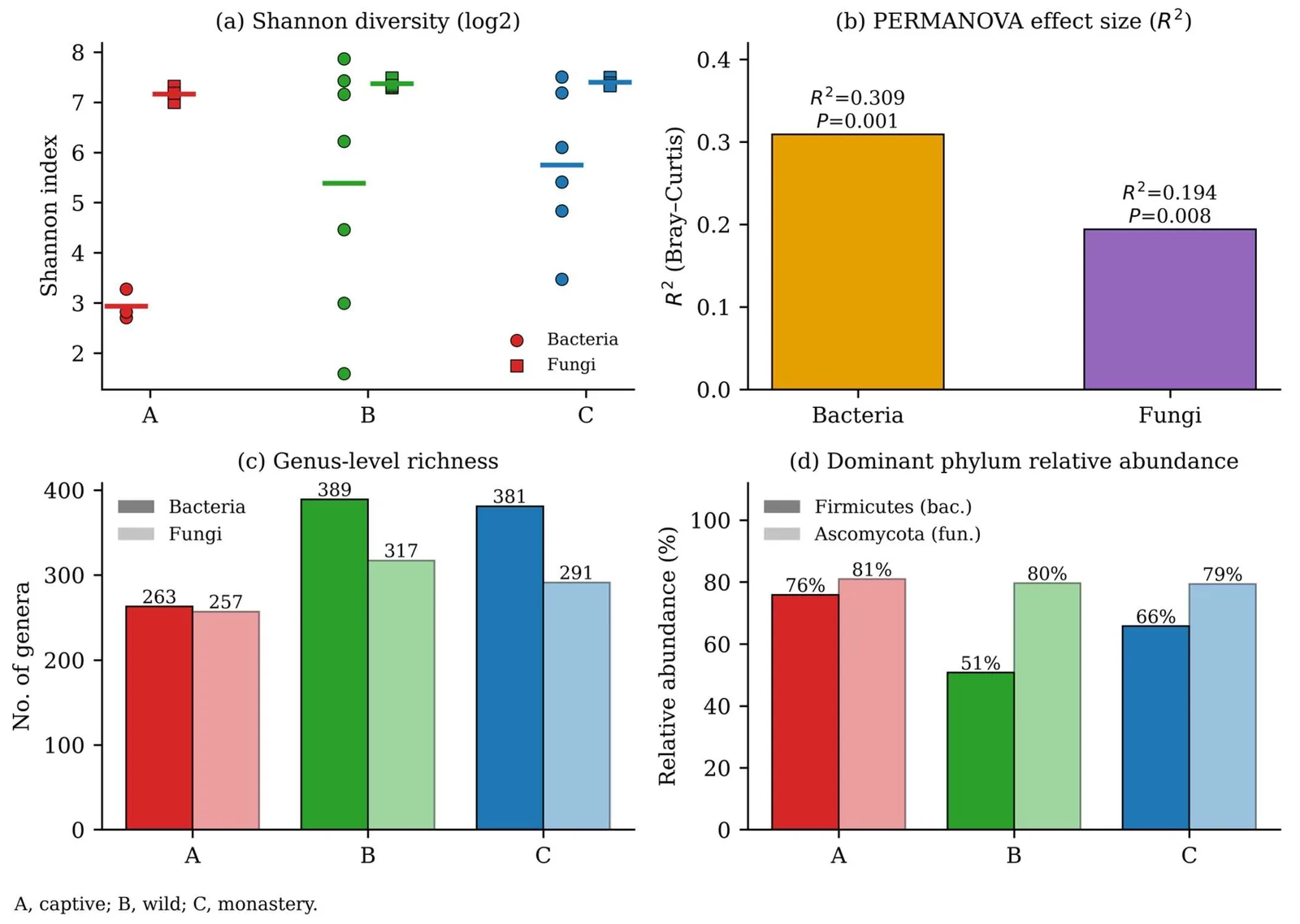
Bacterial and fungal community responses across living conditions. (**a**) Shannon diversity index (log2 basis); (**b**) PERMANOVA effect size (*R*^2^); (**c**) genus-level richness; (**d**) relative abundance of the dominant phylum in each kingdom (Firmicutes for bacteria, Ascomycota for fungi), computed after removal of chloroplast and mitochondrial OTUs. A: fully captive; B: wild; C: semi-wild monastery. PERMANOVA with 9999 permutations.

**Figure 2:**
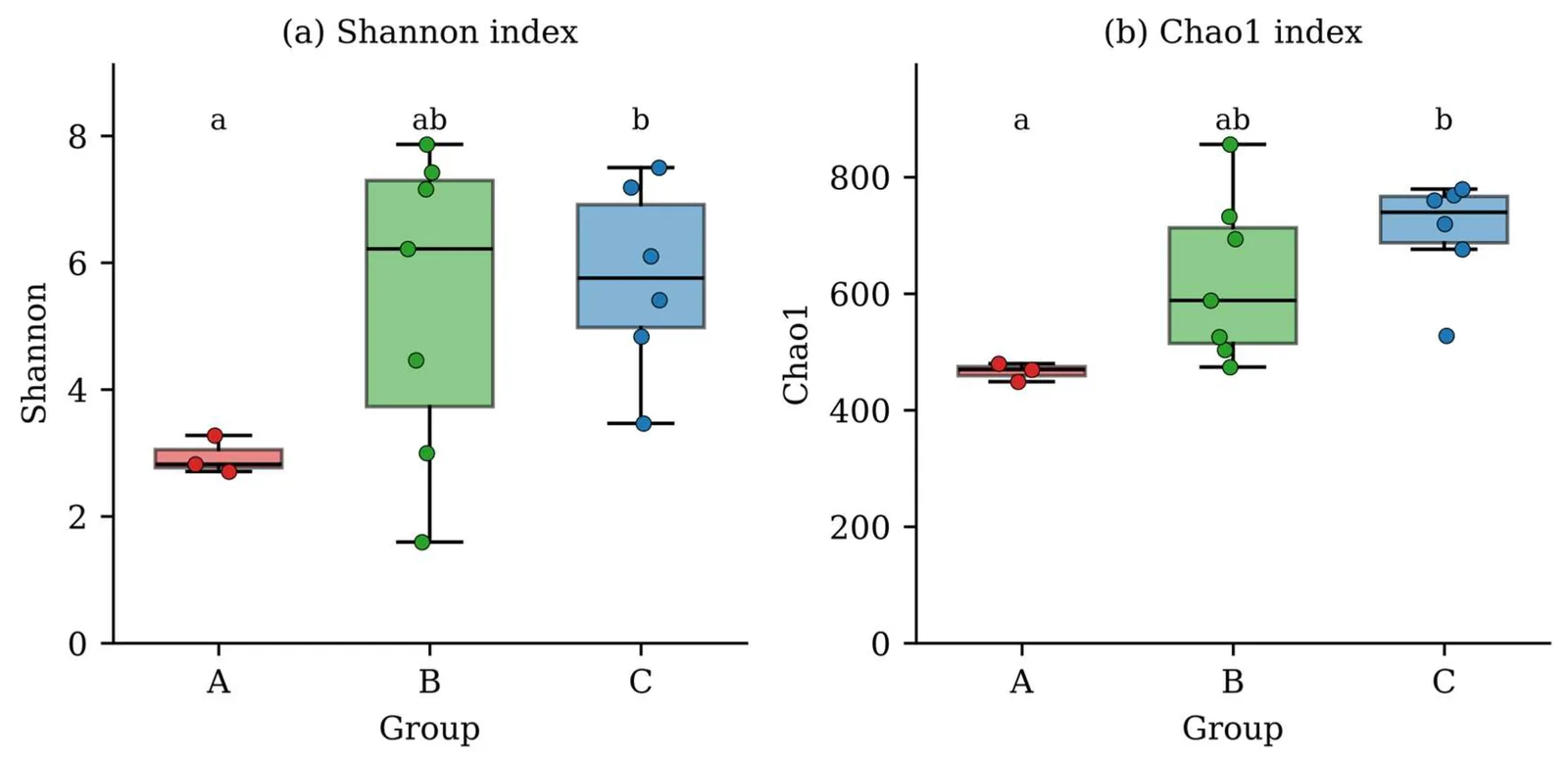
Bacterial alpha diversity across living conditions. (**a**) Shannon index (log2 basis); (**b**) Chao1 index. Groups: A, fully captive (*n* = 3); B, wild (*n* = 7); C, semi-wild monastery (*n* = 6). Different letters indicate significant differences (Tukey HSD, *p* < 0.05). Boxes show the interquartile range and median; whiskers extend to 1.5 × IQR; points represent individual samples.

**Figure 3:**
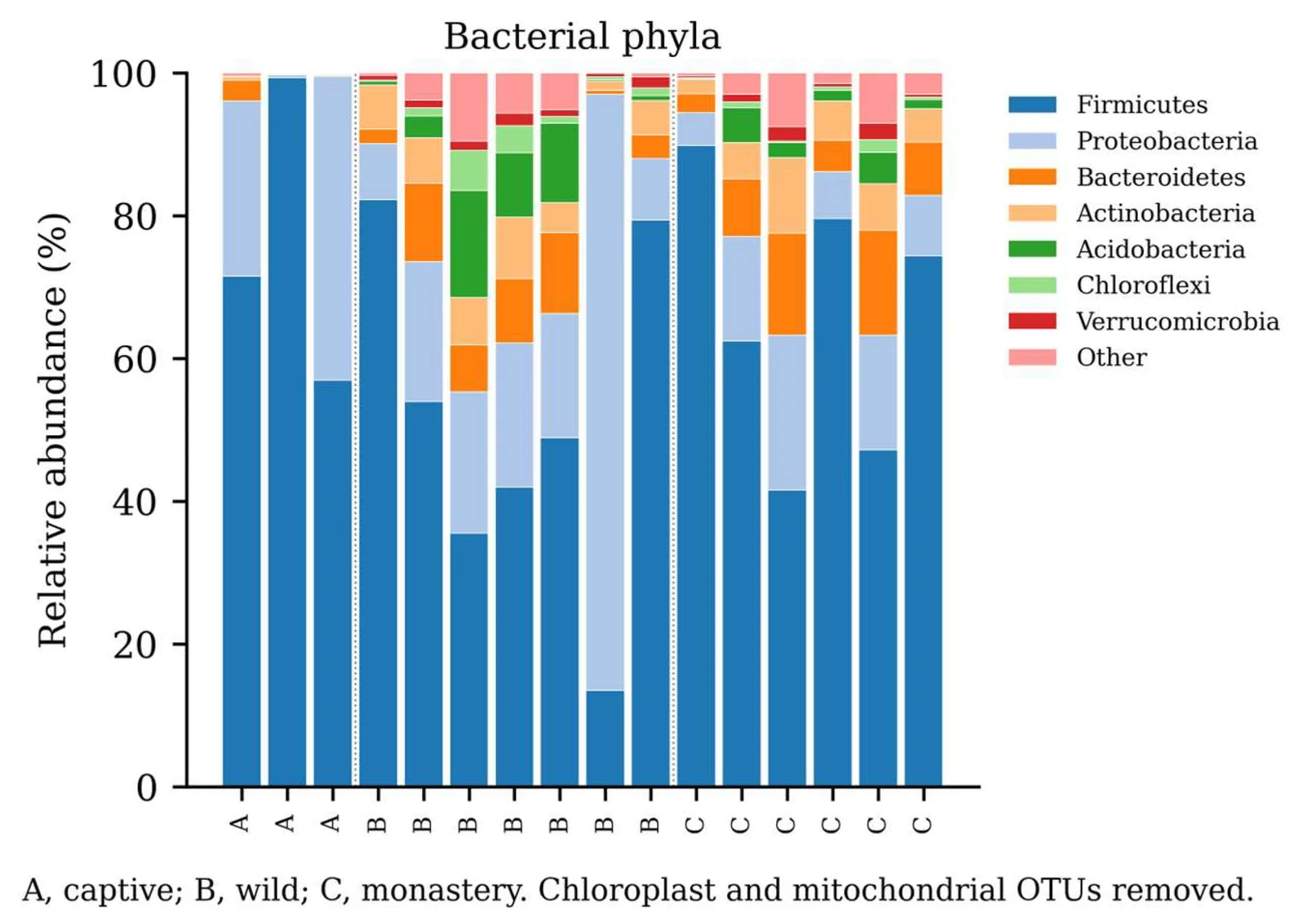
Bacterial phylum composition across living conditions. Chloroplast and mitochondrial OTUs were removed before analysis. A: fully captive; B: wild; C: semi-wild monastery. Bars represent relative abundance (%).

**Figure 4:**
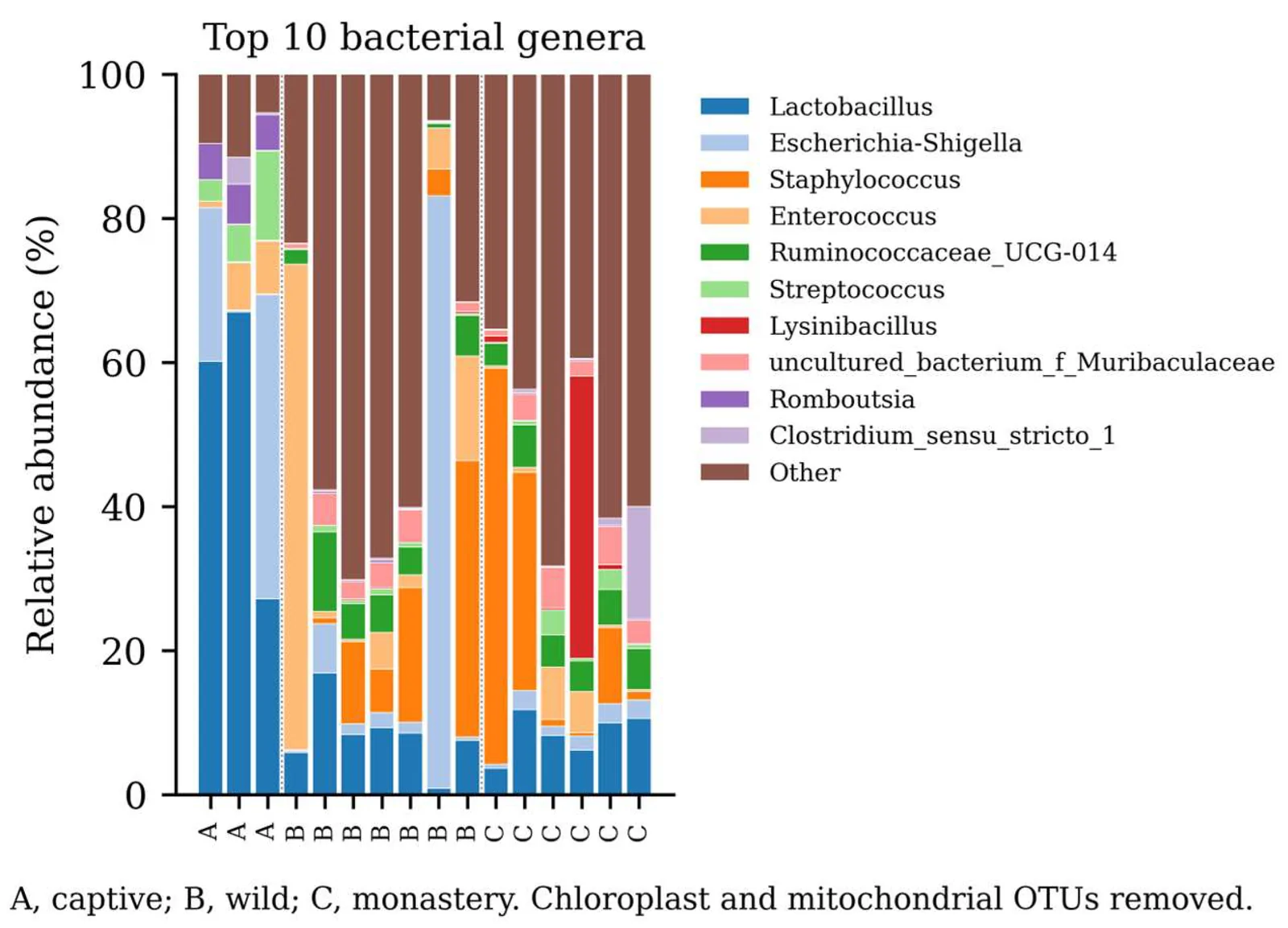
Top bacterial genera across living conditions; chloroplast and mitochondrial OTUs were removed before analysis. Note the extreme *Lactobacillus* dominance in the captive group.

**Figure 5:**
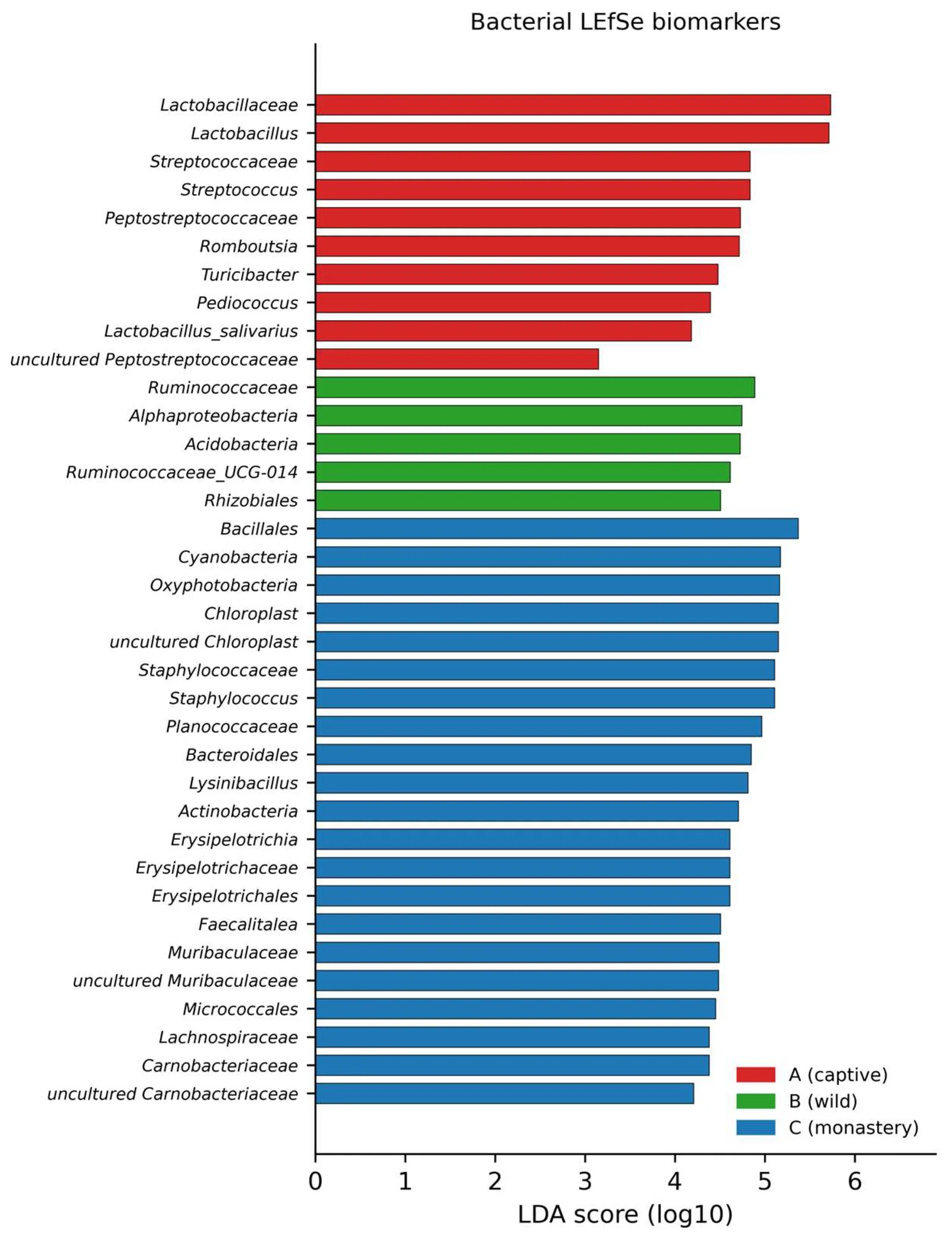
Bacterial taxa enriched in each group (LEfSe). Bars show the linear discriminant analysis (LDA) score (log10) of taxa significantly enriched in each group (biomarker ‘LDA4 less-strict’ analysis, *p* < 0.05), colored by the enriched group: A, captive; B, wild; C, monastery. The complete set of significant taxa is shown in Supplementary Figure S3.

**Figure 6:**
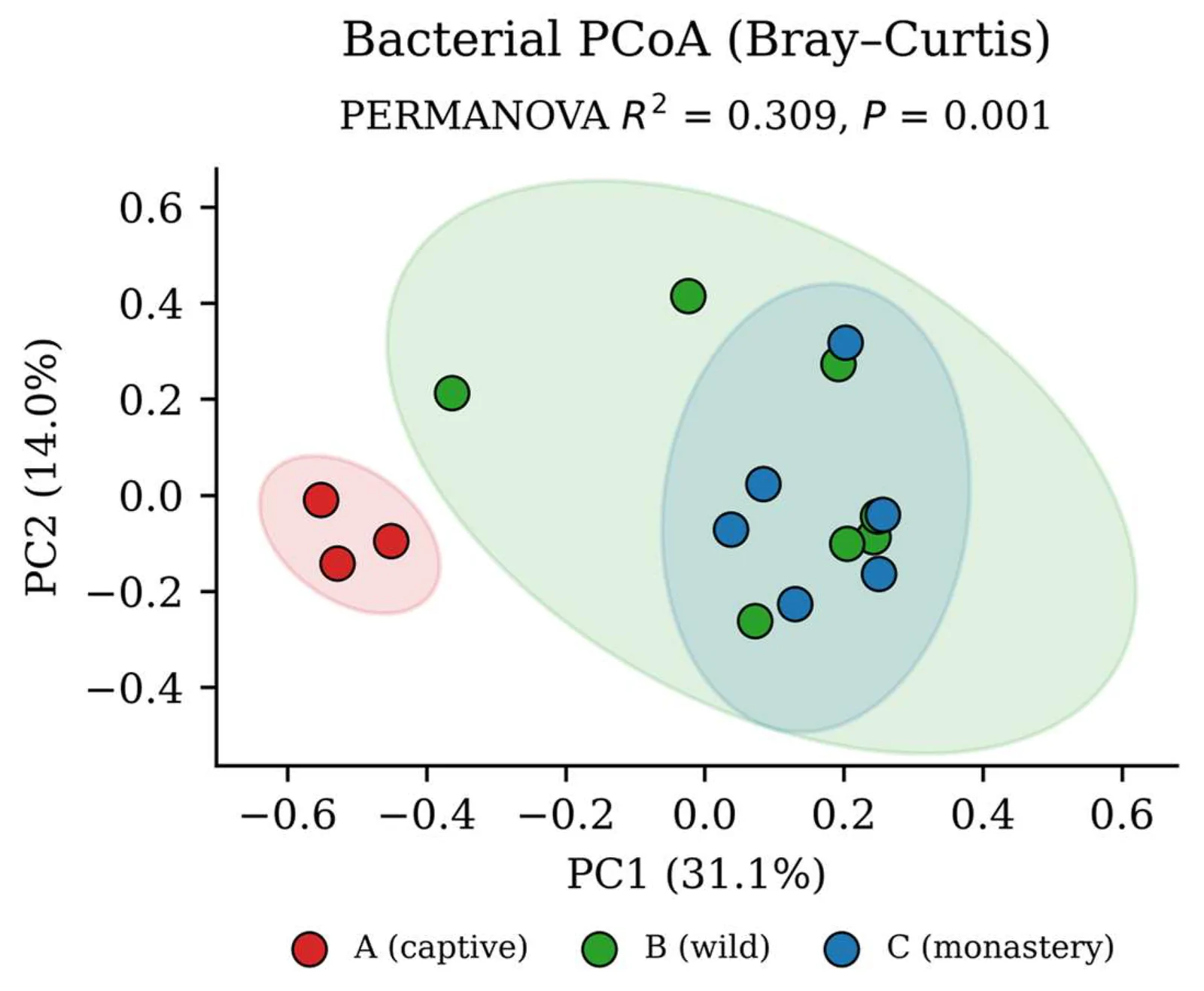
Principal coordinates analysis (PCoA) based on Bray–Curtis distance of gut bacterial communities. Ellipses: 95% confidence intervals. A: fully captive; B: wild; C: semi-wild monastery.

**Figure 7:**
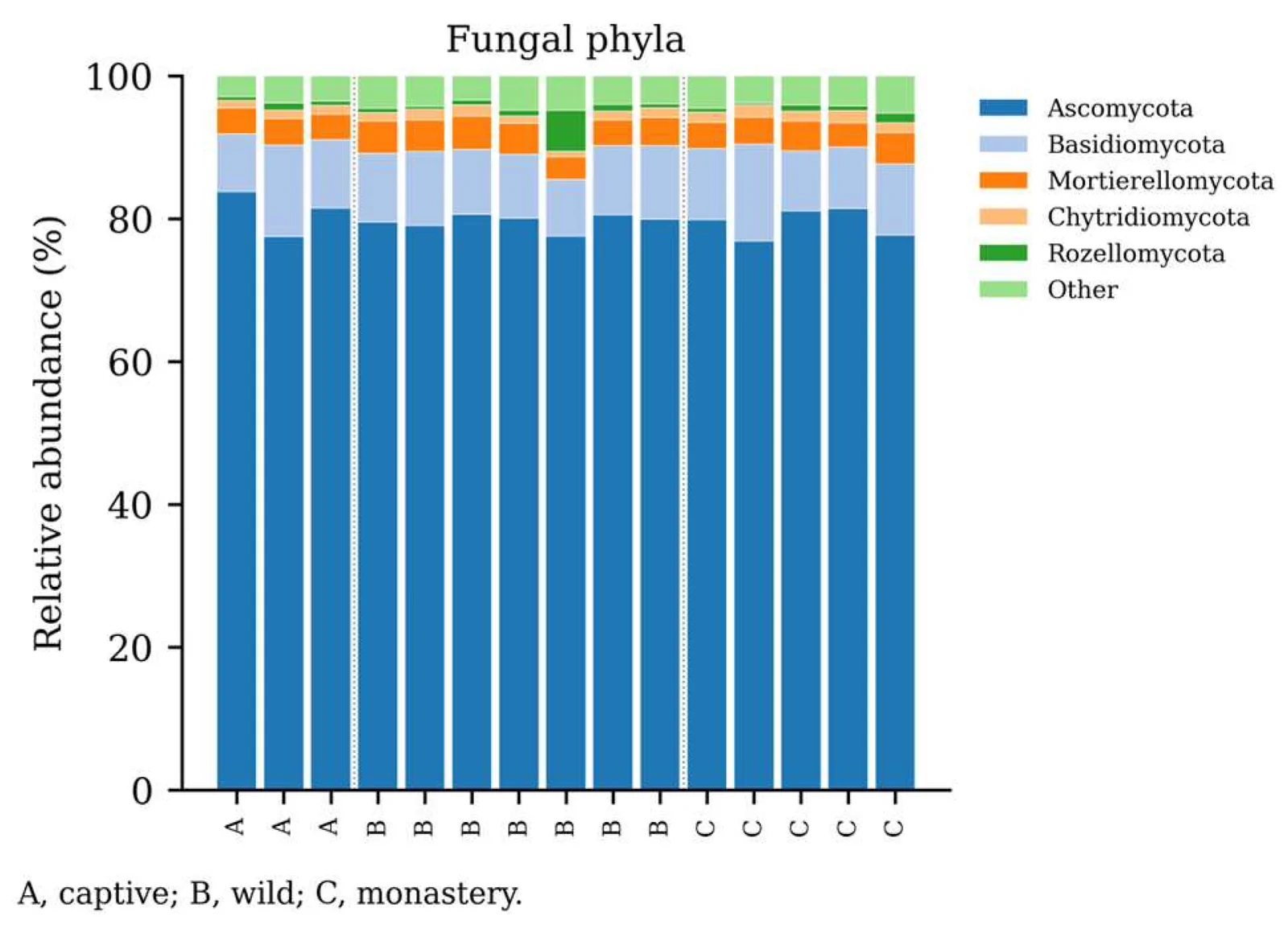
Fungal phylum composition across living conditions. Ascomycota and Basidiomycota dominated all three groups, with only minor differences in phylum-level composition. A: fully captive; B: wild; C: semi-wild monastery.

**Table 1:** PERMANOVA of gut bacterial communities (9999 permutations for the three-group test; for the pairwise contrasts, the number of distinct label partitions is 120 for A vs. B, 84 for A vs. C and 1716 for B vs. C, giving smallest attainable *p*-values of 0.008, 0.012 and 0.0006, respectively); Bray–Curtis and Binary Jaccard distances were computed on the organelle-free OTU table (chloroplast and mitochondrial sequences removed); these two non-phylogenetic metrics are the primary β-diversity metrics. Weighted and Unweighted UniFrac results, which require a reference tree, are shown in Supplementary Figure S2, and the corresponding distance matrices are included in the data deposit. A: fully captive (*n* = 3); B: wild (*n* = 7); C: semi-wild monastery (*n* = 6).

Distance Metric	Comparison	F	R^2^	*p*
Bray–Curtis	Three groups	2.908	0.309	0.001
Binary Jaccard	Three groups	2.905	0.309	<0.001
Bray–Curtis	A vs. B	4.056	0.336	0.008
Binary Jaccard	A vs. B	3.300	0.292	0.008
Bray–Curtis	B vs. C	0.998	0.083	0.423
Binary Jaccard	B vs. C	1.259	0.103	0.158
Bray–Curtis	A vs. C	4.807	0.407	0.012
Binary Jaccard	A vs. C	4.400	0.386	0.012

**Table 2:** PERMANOVA of gut fungal communities (9999 permutations). A: fully captive (*n* = 3); B: wild (*n* = 7); C: semi-wild monastery (*n* = 5).

Distance Metric	Comparison	F	R^2^	*p*
Bray–Curtis	Three groups	1.448	0.194	0.008
Binary Jaccard	Three groups	1.006	0.144	0.447
Bray–Curtis	A vs. B	1.816	0.185	0.016
Binary Jaccard	A vs. B	0.919	0.103	0.866
Bray–Curtis	B vs. C	0.803	0.074	0.943
Binary Jaccard	B vs. C	1.060	0.096	0.241
Bray–Curtis	A vs. C	1.998	0.250	0.018
Binary Jaccard	A vs. C	1.032	0.147	0.386

## Data Availability

Raw FASTQ sequencing data have been deposited in the NCBI Sequence Read Archive under BioProject PRJNA1504888 (BioSamples: SAMN62068666–SAMN62068696; SRA runs: SRR39921735–SRR39921765), publicly accessible at https://www.ncbi.nlm.nih.gov/bioproject/PRJNA1504888. Processed datasets are available at Figshare (https://doi.org/10.6084/m9.figshare.33026111) and include the complete and organelle-free OTU tables, per-sample alpha-diversity tables, taxonomic abundance profiles, the distance matrices used for ordination and PERMANOVA, per-sample metadata and sequencing statistics, the representative OTU sequences and the phylogenetic trees, and the analysis and validation scripts used for the statistical analyses, so that the reported statistical results can be regenerated from the deposited tables. The bacterial LEfSe analysis was run with the sequencing provider’s pipeline; its result table is included in the deposit, so that every LDA score reported here can be checked against the original output. The sequencing quality metrics were likewise produced by the provider and are reported as delivered. All analyses were performed with established, documented software and standard published procedures: USEARCH/UPARSE v10.0 for OTU clustering; the sequencing provider’s standard pipeline for taxonomic assignment, diversity estimation and the LEfSe biomarker analysis; and Python 3.13 with NumPy, SciPy, Matplotlib and NetworkX for the ordination, PERMANOVA, distance-to-centroid dispersion test, distance-based redundancy analysis and rarefaction. Kruskal–Wallis and pairwise Mann–Whitney U tests for differential abundance were computed in the same Python environment. These scripts are deposited with the data, and each implementation follows the standard published formulation of the method it computes; no bespoke statistical method was introduced.

## Supplementary Materials

The following supporting information can be downloaded at https://www.mdpi.com/article/10.3390/ani16213414/s1. Figure S1: leave-one-out sensitivity analysis; Figure S2: PCoA based on Weighted UniFrac; Figure S3: bacterial LEfSe LDA bar chart; Figure S4: bacterial rarefaction curves; Figure S5: bacterial OTU overlap; Figure S6: fungal OTU overlap; Figure S7: genus-level fungal community composition; Figure S8: fungal differential-abundance bar chart; Figure S9: exploratory co-occurrence network; Table S1: organelle read content; Table S2: bacterial OTU counts and alpha diversity; Table S3: rarefaction and elevation sensitivity; Table S4: organelle-free bacterial alpha diversity; Table S5: per-sample sequencing statistics; Table S6: phylum-level bacterial relative abundance; Table S7: genus-level bacterial relative abundance; Table S8: fungal OTU counts and alpha diversity; Table S9: phylum-level fungal relative abundance; Table S10: organelle-free Bray–Curtis PERMANOVA.
