# Supplementary material for: Gut Bacterial and Fungal Communities of the Tibetan Eared Pheasant (Crossoptilon harmani) Across a Captive–Temple–Wild Gradient: Implications for Ex Situ Conservation
Source: Animals (Basel). 2026 Oct 29;16(21):3414. doi: 10.3390/ani16213414 (PMC13761449; doi:10.3390/ani16213414)
Supplement: Supplementary file 1 [file animals-16-03414-s001.zip › animals-4637668-supplementary.pdf]

Supplementary Materials

Supplementary Information

Supplementary Figures S1–S9 and Tables S1–S10 follow. Note on colour conventions: Figures S2–S8 follow the sequencing provider's default palette (Group A, blue; Group B, orange; Group C, green), as stated in the individual captions; Figure S1 and the figures in the main text use red, green and blue for Groups A, B and C, respectively; Figure S9 is coloured by phylum. The two palettes are therefore not interchangeable across figure sets.

I. Supplementary Figures

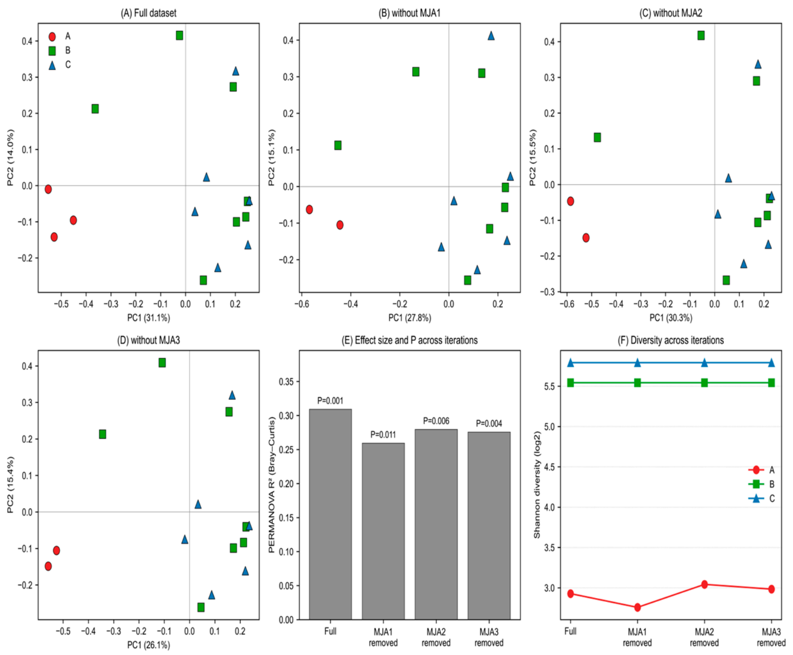

Figure S1. Leave-one-out sensitivity analysis for Group A (n = 3), computed on the organelle-free bacterial table. (A–D) Principal coordinate analysis (PCoA) of Bray–Curtis distances for the full dataset and after removal of each Group A sample; (E) PERMANOVA R^2 and P-values for the full dataset and for each leave-one-out iteration; (F) group mean Shannon diversity (log2) for the full dataset and for each leave-one-out iteration. All three iterations remained significant (P = 0.004–0.011), and no single Group A sample drove the overall pattern.

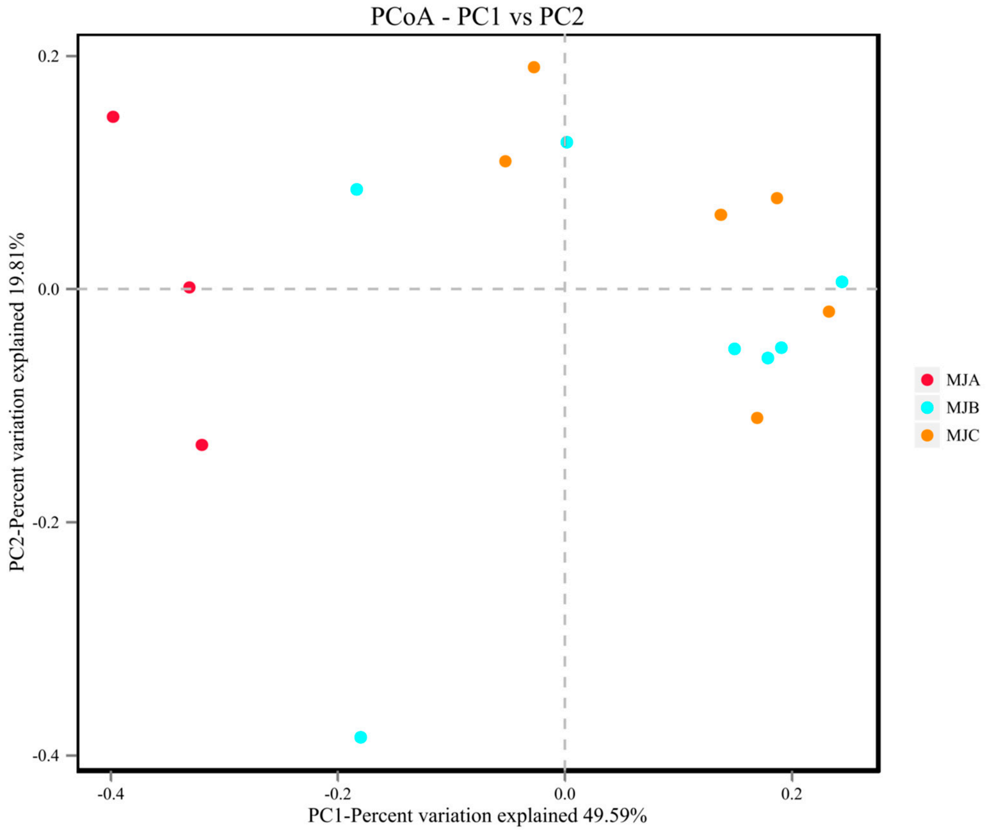

Figure S2. PCoA based on Weighted UniFrac distance of gut bacterial communities. Ellipses: 95% confidence intervals.

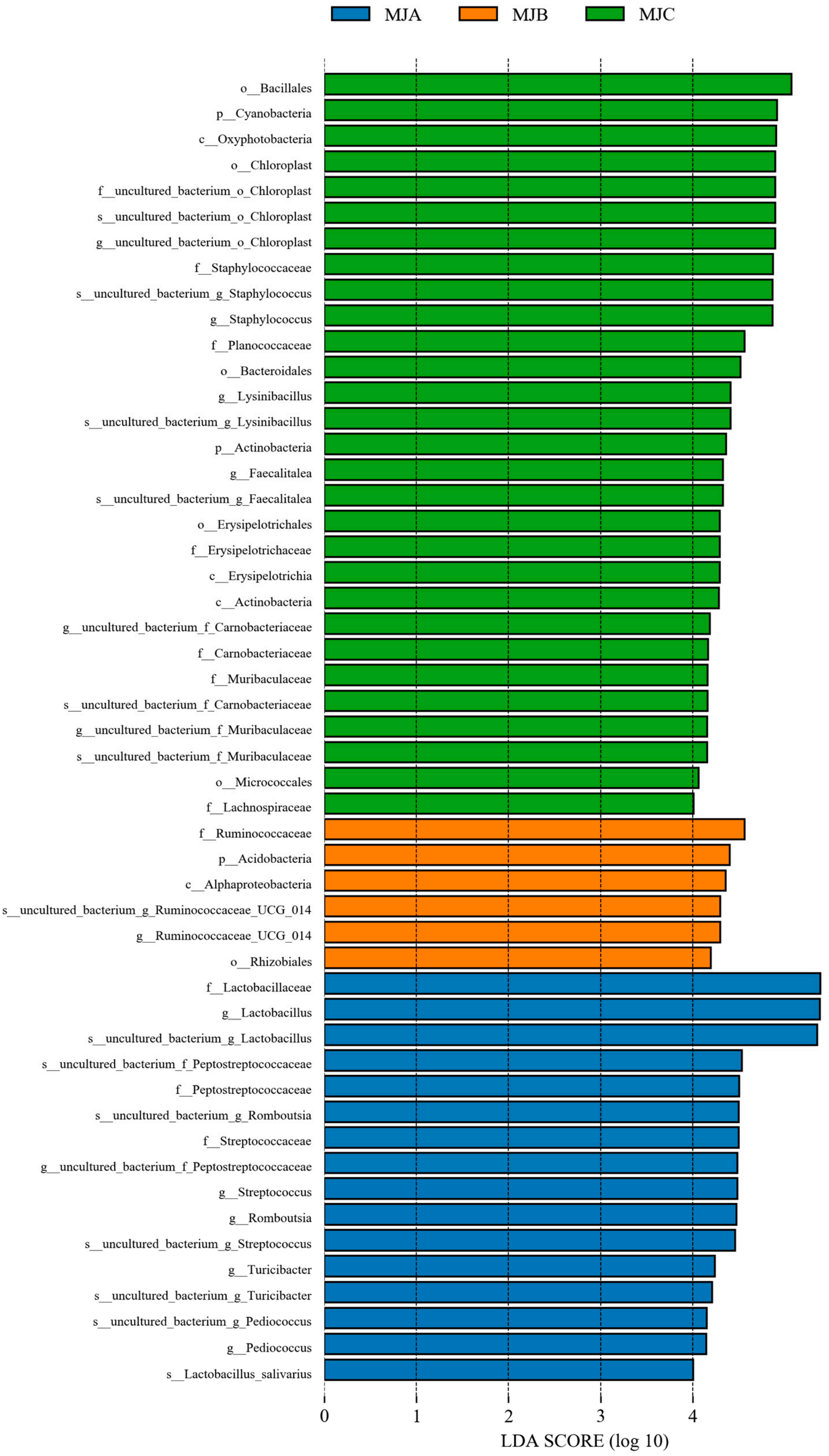

MJA
MJB
MJC
o__Bacillales
p__Cyanobacteria
c__Oxyphotobacteria
o__Chloroplast
f__uncultured_bacterium_o_Chloroplast
s__uncultured_bacterium_o_Chloroplast
g__uncultured_bacterium_o_Chloroplast
f__Staphylococcaceae
s__uncultured_bacterium_g_Staphylococcus
g__Staphylococcus
f__Planococcaceae
o__Bacteroidales
g__Lysinibacillus
s__uncultured_bacterium_g_Lysinibacillus
p__Actinobacteria
g__Faecalitalea
s__uncultured_bacterium_g_Faecalitalea
o__Erysipelotrichales
f__Erysipelotrichaceae
c__Erysipelotrichia
c__Actinobacteria
g__uncultured_bacterium_f_Carnobacteriaceae
f__Carnobacteriaceae
f__Muribaculaceae
s__uncultured_bacterium_f_Carnobacteriaceae
g__uncultured_bacterium_f_Muribaculaceae
s__uncultured_bacterium_f_Muribaculaceae
o__Micrococcales
f__Lachnospiraceae
f__Ruminococcaceae
p__Acidobacteria
c__Alphaproteobacteria
s__uncultured_bacterium_g_Ruminococcaceae_UCG_014
g__Ruminococcaceae_UCG_014
o__Rhizobiales
f__Lactobacillaceae
g__Lactobacillus
s__uncultured_bacterium_g_Lactobacillus
s__uncultured_bacterium_f_Peptostreptococcaceae
f__Peptostreptococcaceae
s__uncultured_bacterium_g_Romboutsia
f__Streptococcaceae
g__uncultured_bacterium_f_Peptostreptococcaceae
g__Streptococcus
g__Romboutsia
s__uncultured_bacterium_g_Streptococcus
g__Turicibacter
s__uncultured_bacterium_g_Turicibacter
s__uncultured_bacterium_g_Pediococcus
g__Pediococcus
s__Lactobacillus_salivarius
0
1
2
3
4
LDA SCORE (log 10)

Figure S3. Bacterial LEfSe LDA bar chart, computed by the sequencing provider on the complete OTU table; the Chloroplast and Cyanobacteria entries therefore represent plant-derived sequences (see Methods). Colors denote groups: blue, Group A; orange, Group B; green, Group C.

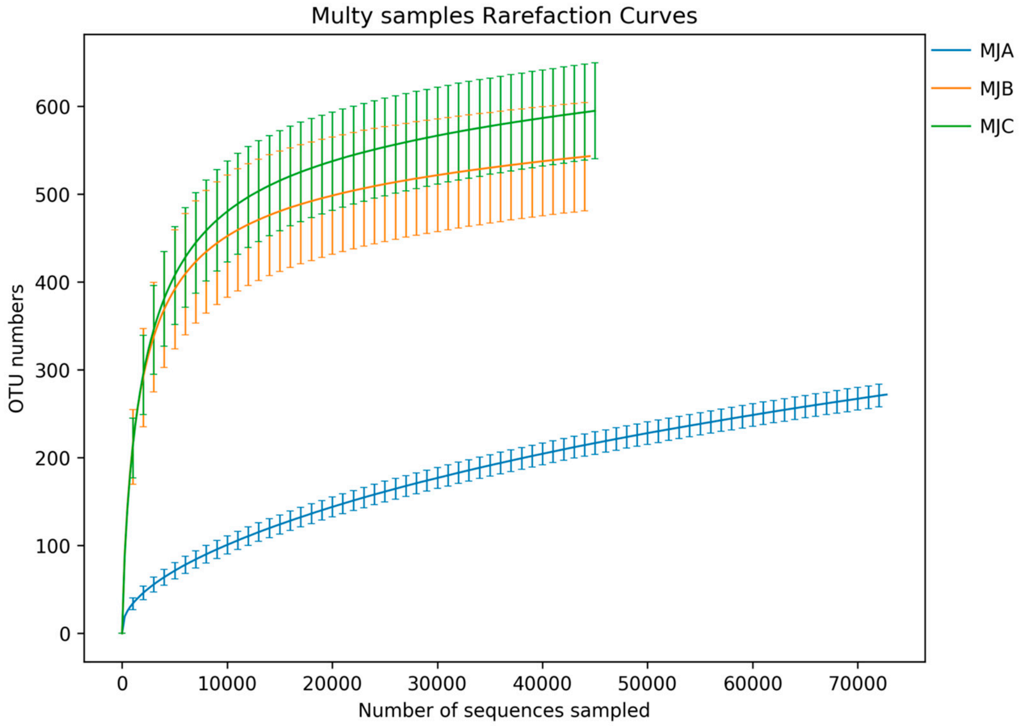

Figure S4. Bacterial rarefaction curves for three groups of Tibetan eared pheasants. Curves approaching plateaus indicate sufficient sequencing depth (Good's coverage > 0.99 in all samples). A: Fully captive (n = 3); B: wild (n = 7); C: semi-wild monastery (n = 6).

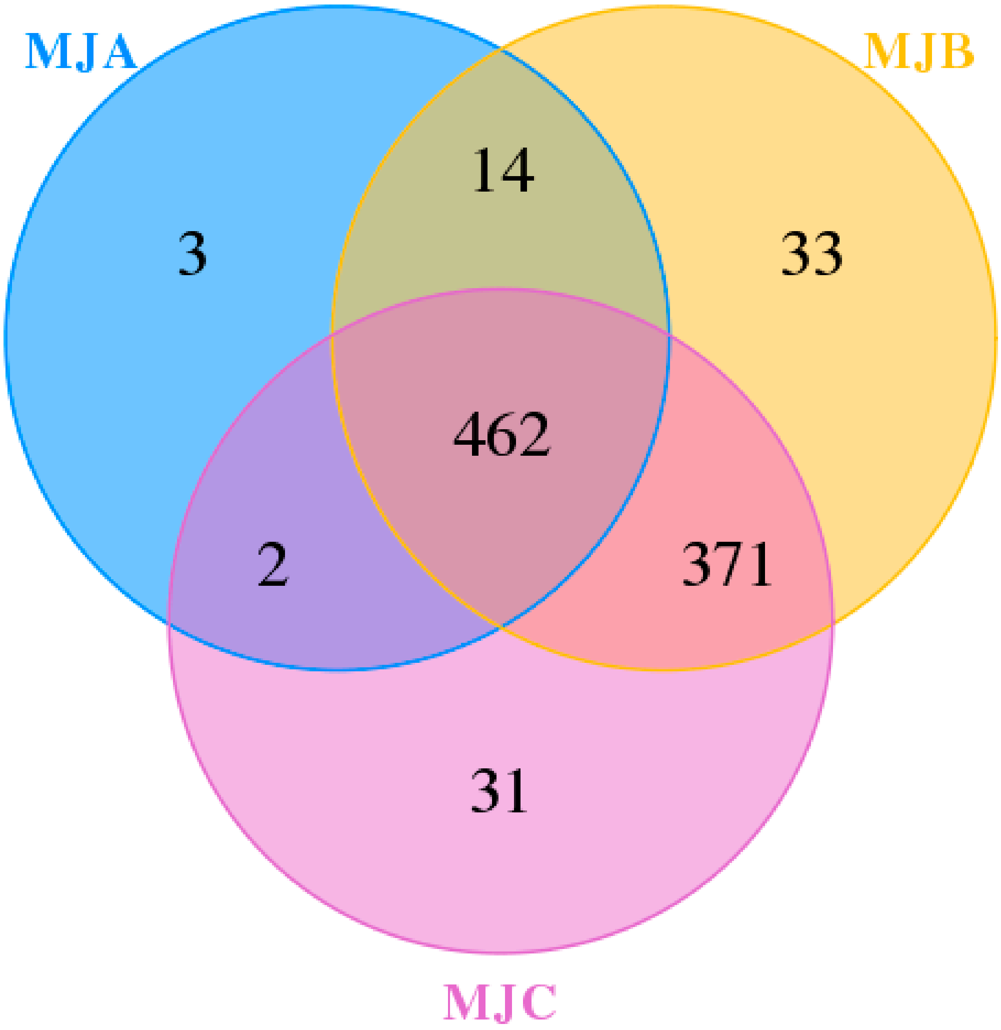

Figure S5. Bacterial OTU overlap among the three groups. A: Fully captive (n = 3); B: wild (n = 7); C: semi-wild monastery (n = 6).

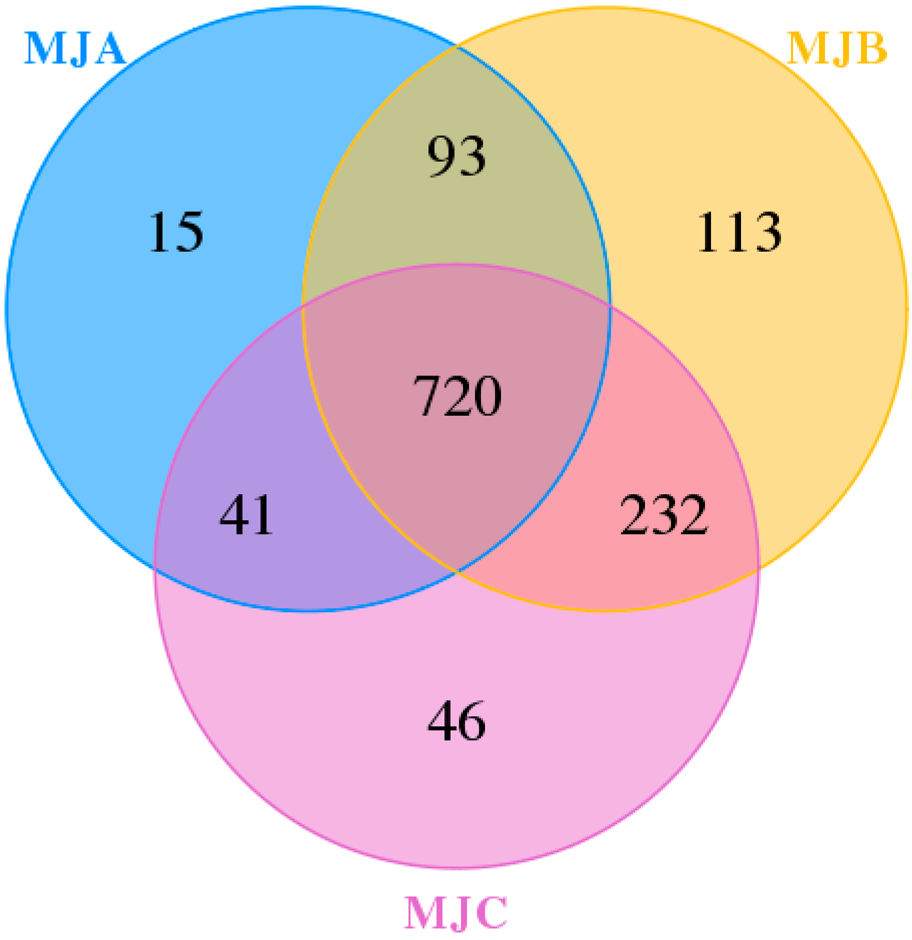

Figure S6. Fungal OTU overlap among the three groups. A: Fully captive (n = 3); B: wild (n = 7); C: semi-wild monastery (n = 5).

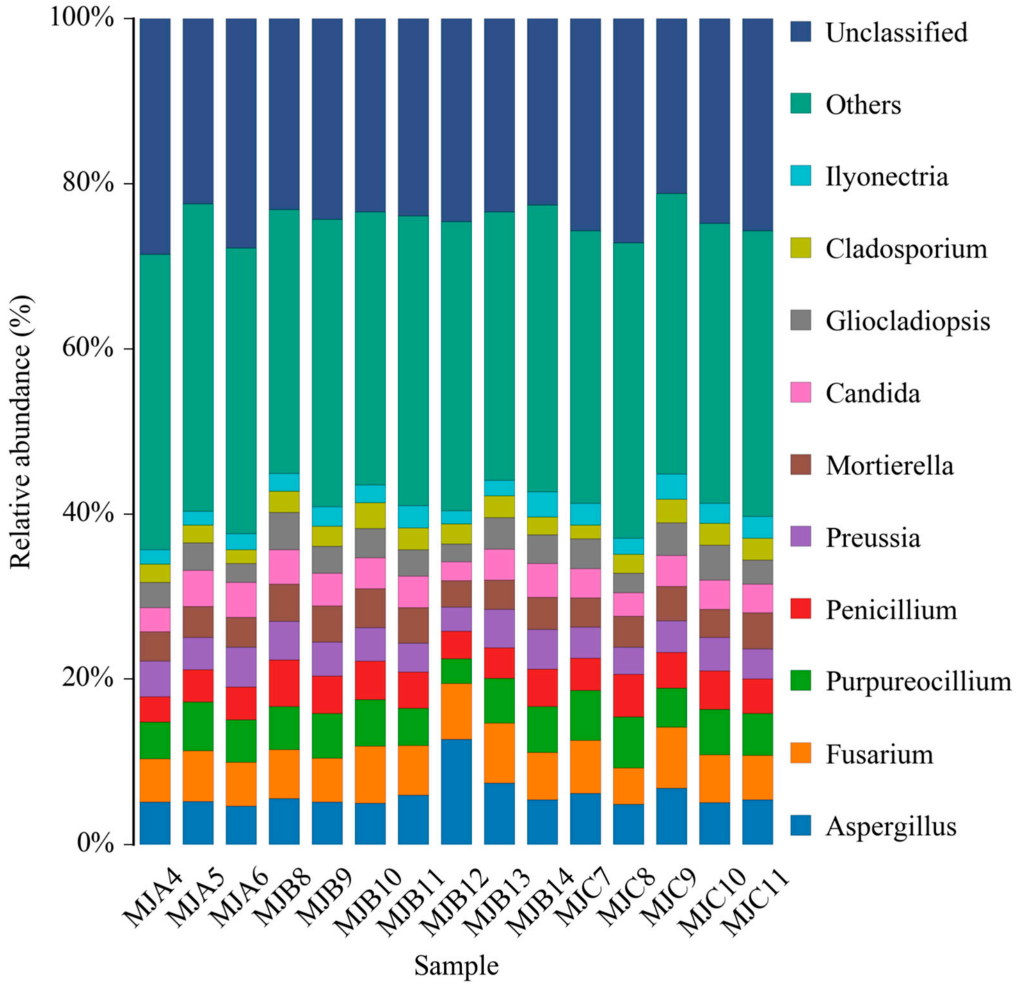

Figure S7. Genus-level gut fungal community composition of Tibetan eared pheasants. A: Fully captive (n = 3); B: wild (n = 7); C: semi-wild monastery (n = 5). No significant differences among groups were detected for the major genera shown here; taxa identified as differentially abundant by the three-group analysis are presented separately in Figure S8.

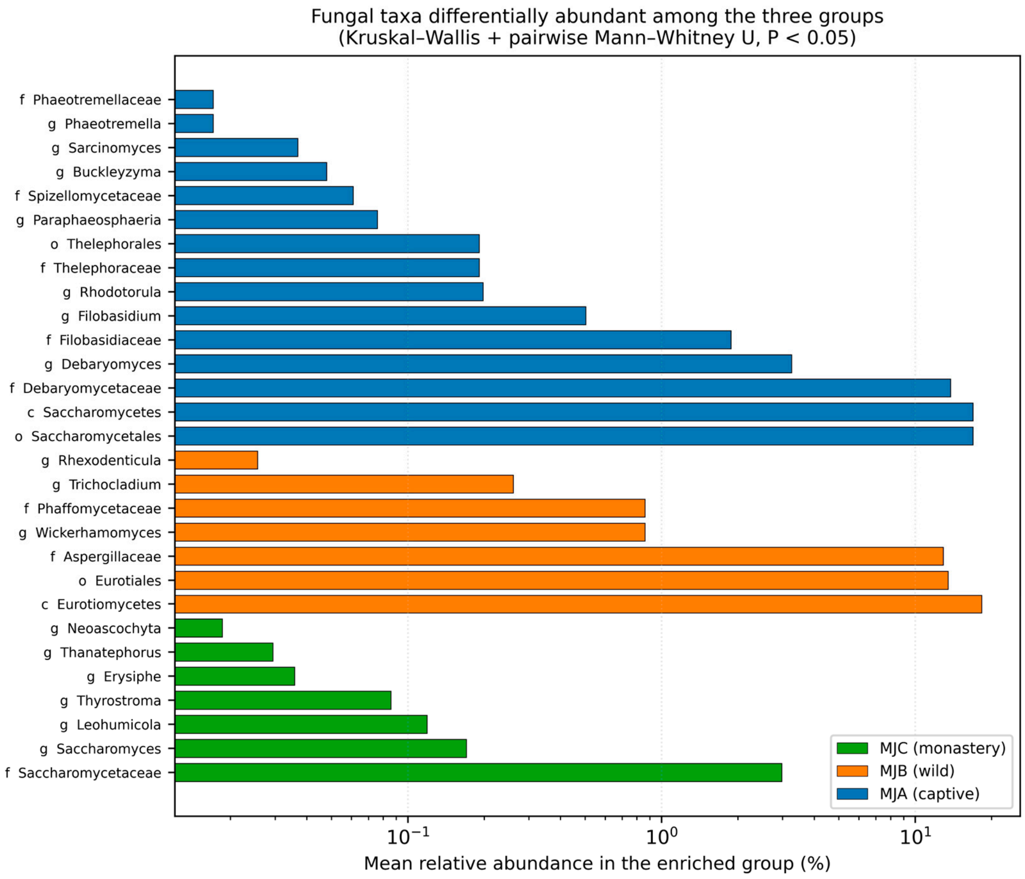

Figure S8. Fungal taxa differentially abundant among the three groups (Kruskal–Wallis with pairwise Mann–Whitney U tests, P < 0.05). Bars show the mean relative abundance of each taxon in the group in which it was enriched; colour denotes the enriched group (blue, Group A; orange, Group B; green, Group C). Analysis performed on the BIOM-formatted OTU table (see Methods).

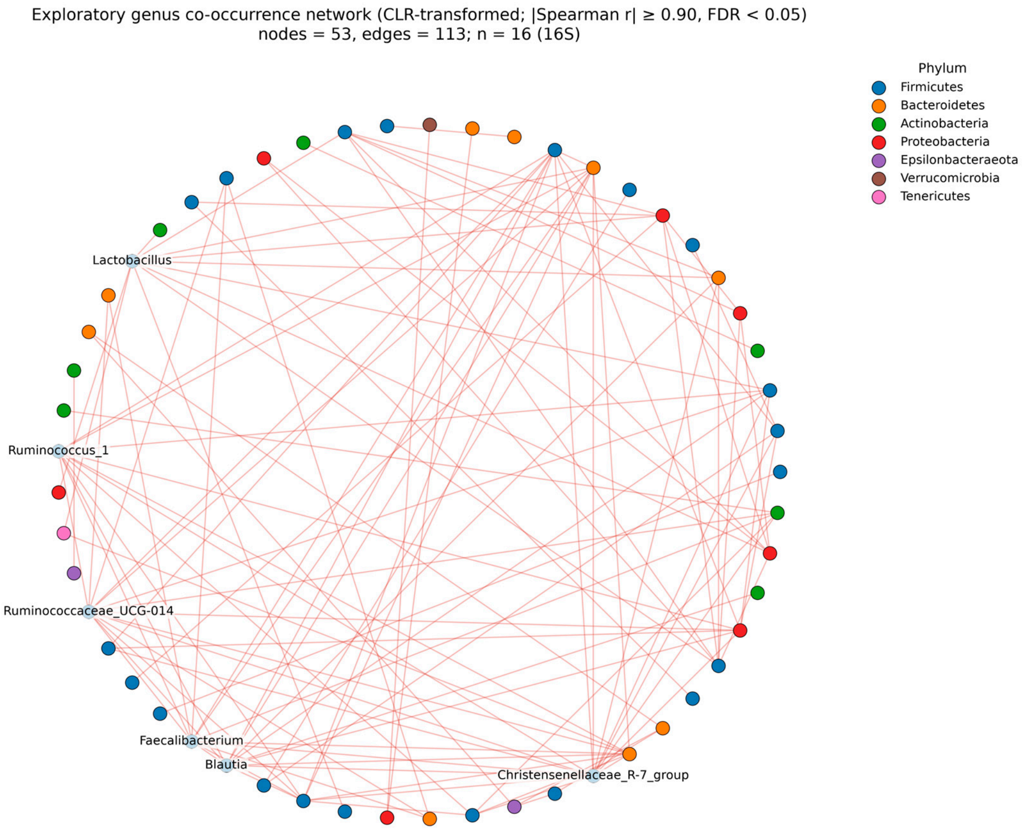

Figure S9. Exploratory genus-level co-occurrence network of the gut bacterial communities (n = 16). The network was built on CLR-transformed genus-level relative abundances, retaining only strong associations (|Spearman r| ≥ 0.90, Benjamini–Hochberg FDR < 0.05); nodes are coloured by phylum and sized by degree. Given the limited sample size, all associations were positive and dominated by taxa that co-vary with the living-condition gradient; the network is therefore presented as an exploratory analysis and is not interpreted as evidence of interspecies interactions.

II. Supplementary Tables

Table S1. Per-sample percentage of reads assigned to chloroplast and mitochondrial OTUs (the plant-derived sequence index discussed in the Methods).

Group	Sample	Library	Chloroplast (%)	Mitochondria (%)
A	MJA1	16S	0.05	0.00
A	MJA2	16S	0.07	0.00
A	MJA3	16S	0.09	0.00
B	MJB1	16S	0.62	0.02
B	MJB2	16S	25.16	0.99
B	MJB3	16S	4.87	0.17
B	MJB4	16S	7.06	0.26
B	MJB5	16S	2.52	0.02
B	MJB6	16S	1.34	0.04
B	MJB7	16S	15.17	0.63
C	MJC1	16S	28.20	0.44

C	MJC2	16S	15.51	0.43
C	MJC3	16S	2.79	0.21
C	MJC4	16S	5.56	0.13
C	MJC5	16S	11.17	0.14
C	MJC6	16S	21.41	0.51

Table S2. Bacterial OTU counts and alpha diversity indices (mean ± SE), computed on the complete OTU table including chloroplast and mitochondrial sequences; organelle-free equivalents are given in Table S4. Shannon values are on a log base 2 scale (multiply by 0.693 for natural logarithms).

Superscript letters indicate Tukey HSD pairwise comparison groupings ($P < 0.05$) computed on the complete OTU table. Three-group ANOVA: OTU richness ($F = 7.63$, $P = 0.006$), Shannon ($F = 2.39$, $P = 0.131$), ACE ($F = 3.38$, $P = 0.065$). Pairwise MJA vs MJC comparisons were significant for Chao1 ($P = 0.017$) and, on the complete table, for Simpson ($P < 0.05$); the Simpson difference was not retained after removal of chloroplast and mitochondrial sequences (Table S4) and is therefore not interpreted as a diversity difference. Letter groupings reflect planned pairwise contrasts and should be interpreted alongside the omnibus test results. ACE: abundance-based coverage estimator.

Index	Group A (Captive, n = 3)	Group B (Wild, n = 7)	Group C (Semi-wild, n = 6)
OTUs	276.33 ± 13.35 a	560.14 ± 55.53 b	609.33 ± 48.73 b
ACE	565.90 ± 48.97	597.49 ± 50.27	663.48 ± 44.96
Chao1	465.92 ± 9.16 a	624.81 ± 53.10 ab	705.31 ± 38.78 b
Simpson	0.7749 ± 0.0280 a	0.7973 ± 0.0959 ab	0.9025 ± 0.0310 b
Shannon	2.9352 ± 0.17 a	5.3898 ± 0.91 ab	5.7514 ± 0.62 b

Table S3. Sensitivity of the bacterial results to sequencing depth, and relationship of community variation to site elevation. (a) Bacterial alpha diversity on tables rarefied to a common depth (39,806 reads per sample; mean of 100 independent rarefactions). (b) Bray–Curtis PERMANOVA on 50 independently rarefied tables (999 permutations each). (c) Fungal Shannon diversity on tables rarefied to 67,334 reads per sample. (d) Variance in the bacterial community (Bray–Curtis, organelle-free) explained by group membership and by elevation, assessed by distance-based redundancy analysis on the positive-eigenvalue PCoA axes. Elevation is a linear function of group membership in this design (the design matrix containing both terms is rank-deficient), so the two cannot be separated by partial analysis.

Index	Group A (n = 3)	Group B (n = 7)	Group C (n = 6)	ANOVA F, P
OTU richness	200.88 ± 12.34	536.17 ± 61.94	590.13 ± 51.82	8.72, P = 0.004
Chao1	367.37 ± 5.53	594.63 ± 59.46	672.82 ± 47.83	5.65, P = 0.017
Shannon (ln)	2.0271 ± 0.12	3.8397 ± 0.67	4.0131 ± 0.47	2.22, P = 0.148
Simpson	0.7745 ± 0.03	0.7966 ± 0.10	0.8774 ± 0.05	0.39, P = 0.683

Comparison	Mean R^2	Mean P	Rarefied datasets with P < 0.05
Three groups	0.309	0.003	50 / 50
A vs B	0.336	0.011	50 / 50
A vs C	0.407	0.016	50 / 50

B vs C	0.083	0.410	0 / 50

Fungal Shannon (ln), rarefied to 67,334 reads	Group A	Group B	Group C	ANOVA
Mean ± SE	4.9668 ± 0.06	5.1110 ± 0.02	5.1278 ± 0.02	F = 6.39, P = 0.013

Model	df	R^2	Adjusted R^2	P
Group (A / B / C)	2	0.309	0.203	0.001
Elevation (linear)	1	0.179	0.121	0.003
Group + Elevation	3	0.309	—	Rank-deficient: elevation is fully nested in group, so no additional variance can be attributed to it

Note: Rarefaction values are means over independent subsamplings; the standard deviation of the group means across rarefactions was < 0.01 for Shannon and Simpson, 4.1 / 1.5 / 1.2 (A / B / C) for OTU richness, and ≤ 26 for Chao1. No negative-control libraries were processed and no partial elevation analysis is possible, as described in the main text.

Table S4. Bacterial alpha diversity after removal of chloroplast and mitochondrial OTUs (mean ± SE). ACE is not reported because the delivered values could not be reproduced by a standard ACE implementation.

Index	Group A (n = 3)	Group B (n = 7)	Group C (n = 6)
OTUs	272.33 ± 13.92	554.57 ± 55.55	602.50 ± 48.82
Chao1	457.92 ± 12.20	619.14 ± 52.79	699.35 ± 38.05
Simpson	0.7746 ± 0.0281	0.7967 ± 0.0999	0.8775 ± 0.0475
Shannon (log2)	2.9276 ± 0.1738	5.5419 ± 0.9692	5.7916 ± 0.6836
Shannon (ln)	2.0292 ± 0.1205	3.8414 ± 0.6720	4.0144 ± 0.4739

Table S5. Per-sample sequencing statistics for gut bacterial (16S rRNA, n = 16) and fungal (ITS1, n = 15) libraries. Group A, fully captive; B, wild; C, semi-wild monastery. Raw reads, clean reads, effective reads and mean read length are given for each sample.

Library	Sample ID	Group	Raw Reads	Clean Reads	Effective Reads	Avg Length (bp)
16S	MJA1	A	79,815	79,560	76,457	427
16S	MJA2	A	80,134	79,900	77,738	427
16S	MJA3	A	80,247	79,982	78,854	428
16S	MJB1	B	80,157	79,868	77,663	424
16S	MJB2	B	80,349	80,060	75,435	415
16S	MJB3	B	79,938	79,661	76,562	419
16S	MJB4	B	80,128	79,818	76,368	418
16S	MJB5	B	79,932	79,688	75,073	418
16S	MJB6	B	79,830	79,552	78,725	428

16S	MJB7	B	79,754	79,496	76,368	420
16S	MJC1	C	79,615	79,286	77,100	421
16S	MJC2	C	71,980	71,693	67,974	419
16S	MJC3	C	79,961	79,659	66,900	419
16S	MJC4	C	80,073	79,797	76,619	423
16S	MJC5	C	80,004	79,671	73,441	418
16S	MJC6	C	79,900	79,622	75,945	416
ITS	MJA4	A	79,779	79,344	78,653	248
ITS	MJA5	A	80,076	79,652	78,952	243
ITS	MJA6	A	79,952	79,499	78,799	246
ITS	MJB8	B	79,943	79,485	78,645	245
ITS	MJB9	B	80,218	79,748	78,960	247
ITS	MJB10	B	80,152	79,695	78,841	245
ITS	MJB11	B	79,914	79,429	78,578	244
ITS	MJB12	B	79,836	79,437	78,508	242
ITS	MJB13	B	79,948	79,502	78,748	243
ITS	MJB14	B	80,017	79,615	78,583	245
ITS	MJC7	C	79,987	79,476	78,681	245
ITS	MJC8	C	79,760	79,284	78,110	256
ITS	MJC9	C	79,866	79,423	78,698	243
ITS	MJC10	C	79,463	78,999	78,078	245
ITS	MJC11	C	80,043	79,610	78,559	247

Table S6. Phylum-level bacterial relative abundance (%, mean ± SE) after removal of chloroplast and mitochondrial OTUs. Groups: A, fully captive (n = 3); B, wild (n = 7); C, semi-wild monastery (n = 6). Cyanobacteria is retained for completeness but decreased to 0.01%, 0.43% and 0.83% in Groups A, B and C once plant-derived chloroplast sequences were removed.

Phylum	Group A	Group B	Group C
Firmicutes	75.91 ± 12.43	50.78 ± 9.16	65.82 ± 7.72
Proteobacteria	22.53 ± 12.22	25.29 ± 9.91	12.00 ± 2.67
Cyanobacteria	0.01 ± 0.00	0.43 ± 0.22	0.83 ± 0.33
Bacteroidetes	1.08 ± 0.93	6.25 ± 1.66	8.62 ± 2.03
Actinobacteria	0.20 ± 0.13	5.44 ± 0.88	5.73 ± 1.15
Acidobacteria	0.05 ± 0.02	5.66 ± 2.26	2.39 ± 0.75
Chloroflexi	0.03 ± 0.03	1.88 ± 0.77	0.61 ± 0.25
Verrucomicrobia	0.01 ± 0.00	1.11 ± 0.17	1.09 ± 0.37

Table S7. Genus-level bacterial relative abundance (%, mean ± SE) after removal of chloroplast and mitochondrial OTUs. Groups as in Table S6. Only the top 10 most abundant genera are shown.

Genus	Group A	Group B	Group C
Lactobacillus	51.44 ± 12.28	8.16 ± 1.81	8.38 ± 1.24
Escherichia–Shigella	21.25 ± 12.15	13.56 ± 11.48	1.93 ± 0.36
Staphylococcus	0.04 ± 0.02	11.31 ± 5.13	16.42 ± 9.02
Enterococcus	4.92 ± 2.05	13.68 ± 9.15	2.43 ± 1.29
Ruminococcaceae_UCG-014	0.11 ± 0.03	4.76 ± 1.25	4.72 ± 0.42

Streptococcus	6.83 ± 2.87	0.48 ± 0.13	1.30 ± 0.58
Lysinibacillus	0.00 ± 0.00	0.10 ± 0.04	6.89 ± 6.47
Uncultured Muribaculaceae	0.03 ± 0.01	2.43 ± 0.68	3.45 ± 0.76
Romboutsia	5.20 ± 0.22	0.16 ± 0.04	0.16 ± 0.02
Clostridium_sensu_stricto_1	1.34 ± 1.17	0.12 ± 0.04	2.91 ± 2.55

Table S8. Fungal OTU counts and alpha diversity indices (mean ± SE). Shannon values are on a log base 2 scale. OTU numbers approach saturation at the sequencing depth obtained, so the Chao1 and ACE values, which extrapolate to unobserved taxa, should be interpreted with caution. Groups: A, fully captive; B, wild; C, semi-wild monastery.

Index	Group A (n = 3)	Group B (n = 7)	Group C (n = 5)
OTUs	558.33 ± 9.13	584.43 ± 26.16	564.20 ± 9.48
ACE	578.69 ± 7.98	625.35 ± 24.76	610.96 ± 25.41
Chao1	602.97 ± 18.61	667.20 ± 22.28	632.56 ± 15.24
Simpson	0.9811 ± 0.0030	0.9852 ± 0.0008	0.9863 ± 0.0005
Shannon	7.1662 ± 0.09	7.3741 ± 0.03	7.3983 ± 0.03

Table S9. Phylum-level fungal relative abundance (%, mean ± SE). Groups as in Table S8.

Phylum	Group A	Group B	Group C
Ascomycota	80.90 ± 1.83	79.60 ± 0.40	79.40 ± 0.91
Basidiomycota	10.20 ± 1.39	9.42 ± 0.33	10.10 ± 0.92
Mortierellomycota	3.65 ± 0.03	4.10 ± 0.20	3.89 ± 0.18
Chytridiomycota	1.11 ± 0.05	1.22 ± 0.11	1.50 ± 0.08

Table S10. Bray–Curtis PERMANOVA after removal of chloroplast and mitochondrial OTUs (9,999 permutations).

Comparison	F	R^2	P
Three groups	2.908	0.309	0.001
A vs B	4.056	0.336	0.008
A vs C	4.807	0.407	0.012
B vs C	0.998	0.083	0.423

Note: One-way ANOVA on organelle-free Shannon (log2) F = 2.22, P = 0.148; Kruskal–Wallis H = 3.83, P = 0.147; OTU richness ANOVA F = 7.49, P = 0.007 (Tukey HSD: A vs B P = 0.015, A vs C P = 0.007, B vs C P = 0.773); Chao1 ANOVA F = 4.70, P = 0.029 (Tukey HSD A vs C P = 0.023); Simpson ANOVA F = 0.39, P = 0.683. Chloroplast and mitochondrial OTUs accounted for 8.55% and 0.24% of all reads, respectively (8.79% combined), and were the only OTUs removed (909 of 916 retained).
